# A Crystal Structure of the Dengue Virus NS5 Protein Reveals a Novel Inter-domain Interface Essential for Protein Flexibility and Virus Replication

**DOI:** 10.1371/journal.ppat.1004682

**Published:** 2015-03-16

**Authors:** Yongqian Zhao, Tingjin Sherryl Soh, Jie Zheng, Kitti Wing Ki Chan, Wint Wint Phoo, Chin Chin Lee, Moon Y. F. Tay, Kunchithapadam Swaminathan, Tobias C. Cornvik, Siew Pheng Lim, Pei-Yong Shi, Julien Lescar, Subhash G. Vasudevan, Dahai Luo

**Affiliations:** 1 Program in Emerging Infectious Diseases, DUKE-NUS Graduate Medical School, Singapore; 2 NUS Graduate School for Integrative Sciences and Engineering, National University of Singapore, Singapore; 3 School of Biological Sciences, Nanyang Technological University, Singapore; 4 Novartis Institute for Tropical Diseases, Singapore; 5 Lee Kong Chian School of Medicine, Nanyang Technological University, Singapore; 6 Department of Biological Sciences, National University of Singapore, Singapore; 7 UPMC UMRS CR7—CNRS ERL 8255-INSERM U1135 Centre d’Immunologie et des Maladies Infectieuses, Centre Hospitalier Universitaire Pitié-Salpêtrière, Faculté de Médecine Pierre et Marie Curie, Paris, France; Institut Pasteur, FRANCE

## Abstract

Flavivirus RNA replication occurs within a replication complex (RC) that assembles on ER membranes and comprises both non-structural (NS) viral proteins and host cofactors. As the largest protein component within the flavivirus RC, NS5 plays key enzymatic roles through its N-terminal methyltransferase (MTase) and C-terminal RNA-dependent-RNA polymerase (RdRp) domains, and constitutes a major target for antivirals. We determined a crystal structure of the full-length NS5 protein from Dengue virus serotype 3 (DENV3) at a resolution of 2.3 Å in the presence of bound SAH and GTP. Although the overall molecular shape of NS5 from DENV3 resembles that of NS5 from Japanese Encephalitis Virus (JEV), the relative orientation between the MTase and RdRp domains differs between the two structures, providing direct evidence for the existence of a set of discrete stable molecular conformations that may be required for its function. While the inter-domain region is mostly disordered in NS5 from JEV, the NS5 structure from DENV3 reveals a well-ordered linker region comprising a short 3_10_ helix that may act as a swivel. Solution Hydrogen/Deuterium Exchange Mass Spectrometry (HDX-MS) analysis reveals an increased mobility of the thumb subdomain of RdRp in the context of the full length NS5 protein which correlates well with the analysis of the crystallographic temperature factors. Site-directed mutagenesis targeting the mostly polar interface between the MTase and RdRp domains identified several evolutionarily conserved residues that are important for viral replication, suggesting that inter-domain cross-talk in NS5 regulates virus replication. Collectively, a picture for the molecular origin of NS5 flexibility is emerging with profound implications for flavivirus replication and for the development of therapeutics targeting NS5.

## Author Summary

DENV causes widespread mosquito-borne viral infections worldwide and nearly 40% of the world’s population is at risk of being infected. Currently, no licensed vaccines or specific drugs are available to treat severe infections by DENV. NS5 is a large protein of 900 amino acids composed of two domains with several key enzymatic activities for viral RNA replication in the host cell and constitutes a prime target for the design of antiviral inhibitors. We succeeded in trapping a stable conformation of the full-length NS5 protein and report its crystal structure at a resolution of 2.3 Å. This conformation reveals the entire inter-domain region and clarifies the determinants of NS5 flexibility. The inter-domain interface is stabilized by several polar contacts between residues projecting from the MTase and RdRp domains of NS5. Several evolutionarily conserved residues at the interface play a crucial role for virus replication as shown by reverse genetics, although the analogous mutations mostly do not abolish the *in vitro* enzymatic activities of the recombinant proteins.

## Introduction

Several flaviviruses such as Dengue virus (DENV), Japanese Encephalitis virus (JEV), West Nile virus (WNV), Yellow Fever virus (YFV) and Tick-Borne Encephalitis virus (TBEV) are major human pathogens. The mosquito-borne DENV serotypes 1–4 cause widespread epidemics and nearly 40% of the world’s population is at risk of being infected [1]. Infection by any of the four serotypes can lead to a broad spectrum of outcomes, ranging from asymptomatic infection, dengue fever, dengue hemorrhagic fever or dengue shock syndrome. A tetravalent vaccine is undergoing phase III of clinical trials, which requires three booster injections and only confers partial cross protection. No antivirals have been approved to treat Dengue so far, although availability of such molecules would be valuable to treat dengue infection.

Flavivirus RNA replication occurs within a multi-protein replication complex (RC), which assembles on ER-derived membranes and comprises both non-structural (NS) viral proteins and host cofactors [2–4]. With 900 amino acid residues, NS5 is the largest enzyme and the most conserved protein component of the flavivirus RC. Its N-terminal domain (residues 1–262 in DENV3) belongs to the S-adenosyl-L-methionine (SAM)-dependent methyltransferase (MTase) superfamily [5]. The MTase domain of NS5 caps the viral RNA genome, a step required for its stability and translation into viral polyproteins by host cell ribosomes [6]. Methylations of the N7 atom of Guanine-0, the 2’-*O* atoms of the ribose of Adenosine-1 and internal adenosines also contribute to viral escape from the host cell innate immune response [5] [7] [8] [9]. A putative guanylyltransferase activity (GTase) was also proposed for the N-terminal domain of NS5 [10,11] but this notion remains debatable. The C-terminal domain (residues 273–900) of NS5 contains the RNA-dependent RNA polymerase (RdRp) that synthesizes the anti-genome and progeny genome [12,13]. The RdRp domain is composed of the Finger, Thumb and Palm subdomains that are structurally conserved across viral RdRps [13]. Within the RdRp domain, residues 316–415 contain functional nuclear localization sequences that are hotspots for interactions with other viral and host proteins [14–17]. NS5 interacts with the NS3 protease-helicase and with several host proteins, including components of the ubiquitin proteasome pathway, NF90, and eEF1A [17–20]. In addition to its enzymatic functions required for RNA replication, NS5 acts as an antagonist of the host interferon response by interacting with and promoting the degradation of STAT2 [21]. In DENV, NS5 localizes to the nucleus of infected cells in a regulated and serotype-dependent manner that may modulate other host processes [22,23]. The importance of NS5 in viral replication and host immune response modulation makes it an ideal target for developing broad-acting antiviral inhibitors to treat diseases caused by flaviviruses [24–28]. A putative linker (or “inter-domain region” spanning residues 263–272) connects the two catalytic domains of NS5. Its sequence is relatively poorly conserved although its length has been preserved across flaviviruses.

Structural and functional studies of the complete NS5 polypeptide have been initiated by several research groups. Using *in silico* docking guided by reverse genetics, a first model for NS5 from WNV was proposed [12]. In this model, the MTase active site was placed in an orientation that would facilitate the capping of newly synthesized viral RNA exiting from the template-binding channel of the RdRp domain. The conformation of NS5 from DENV3 was also studied using small angle X-ray scattering (SAXS) [29] which suggested that the isolated NS5 protein from DENV may be flexible and could adopt a range of conformations, from compact (80% of the population) to more extended (20% of the population) structures in solution [29]. It was also reported that the MTase domain of NS5 does not affect its RdRp activity and that the two domains of NS5 responsible for RNA capping and synthesis respectively function essentially independently [30]. Recently, Lu and Gong reported a crystal structure for NS5 from JEV, in which the MTase and RdRp domains adopt a compact conformation stabilized by an interface dominated by hydrophobic interactions [31]. Moreover, a fragment of the RdRp from DENV3 NS5 containing extra residues from the linker has enhanced thermo-stability and polymerase activity, suggesting an impact of residues N-terminal to the polymerase domain on RdRp activity and stability [32]. This observation is supported by a recent study on viral RNA synthesis demonstrating that DENV2 NS5 has superior *de novo* and elongation activities than the NS5-RdRp domain and proposing that the MTase domain interacts with RdRp domain dynamically to regulate RNA synthesis during virus replication [33]. Clearly, more experimental data are needed to clarify the potential cross-regulatory effects between the two enzymatic domains of NS5, to reveal its distinct conformations, the molecular origin of its flexibility and to understand how its dynamic properties relate to the various steps required for RNA capping and synthesis. Here, we crystallized the full length NS5 protein from DENV3 and determined its crystal structure at 2.3 Å resolution, bound to SAH and also in the presence of GTP. Although the overall molecular shape of NS5 from DENV3 is similar to the NS5 protein from JEV, the relative orientation between their MTase and RdRp domains differs markedly between the two structures. The crystal structure of NS5 from DENV3 reveals a well-ordered linker region and a unique and mostly polar interface between the MTase and RdRp domains. The importance of selected interface residues in the structure and function of NS5 was addressed by site-directed mutagenesis for biochemical and virus replication studies. These studies corroborate the structural insight that inter-domain interactions are critical for viral RNA replication and infection.

## Materials and Methods

### Cells

BHK-21 cells (Baby Hamster Kidney fibroblast cells, ATCC) were maintained in RPMI 1640 media (Gibco), supplemented with 10% fetal bovine serum (FBS) and 1% penicillin/streptomycin (P/S) at 37°C in 5% CO_2_.

### Cloning, expression and purification of DENV3 NS5 for crystallization

To facilitate protein crystallization, we removed five flexible amino acids each from the N- and C- terminal ends of NS5, based on inspection of missing electron density in the structures of the individual MTase and RdRp domains from DENV3 (PDB codes 3P97 and 2J7U). The fragment corresponding to amino acid residues 6 to 895 of DENV3 NS5 (GenBank accession number AY662691.1) was amplified and cloned into pNIC28-Bsa4 plasmid using ligation independent cloning methods [34]. The expressed construct comprises the hexa-histidine tag and a Tobacco Etch Virus (TEV) protease cleavage site fused at the N terminus of DENV3 NS5. Transformed *E*. *coli* BL21-CodonPlus (Stratagene) was grown in LB medium containing 50 μg/ml kanamycin and 34 ug/ml chloramphenicol to an A_600nm_ of 0.6–0.8 at 37°C. Expression of the recombinant proteins was induced by the addition of 0.5 mM isopropyl β-D-galactoside, and incubation was continued for a further 20 h at 18°C. The cells were rapidly cooled and harvested by centrifugation at 8000 g for 10 min at 4°C and stored at -20°C. Cells were resuspended in buffer A (20 mM Tris-HCl, pH 7.5, 500 mM NaCl, 5mM β-mercaptoethanol, 10% glycerol, 10 mM imidazole) and lysed by sonication. The lysate was clarified by centrifugation at 20,000 g for 60min at 4°C. The supernatant was purified by nickel-nitrilo-triacetic acid affinity chromatography by washing unbound protein with buffer A supplemented with 40 mM imidazole. The DENV3 NS5 protein was eluted using a linear gradient of imidazole ranging from 40 to 500 mM. Fractions containing His_6_-NS5 were dialyzed against buffer C (20 mM Na-Hepes, pH 7.5, 300 mM NaCl, 5 mM DTT, 10% (v/v) glycerol), and the proteins were cleaved by TEV (substrate-to-enzyme ratio of 40:1, w/w) at 4°C for overnight. NS5 was further purified by size-exclusion chromatography using a HiPrep Superdex-200 gel filtration column (Amersham Bioscience) in buffer C. Fractions containing NS5 were pooled and concentrated to ~10 mg/ml before storage in -80°C. SDS-PAGE analysis of the resulting NS5 protein indicated a purity of 95%.

### Crystallization and data collection

Crystallization trials were set up at 20°C with a Phoenix crystallization robot using sitting drop vapour diffusion. After extensive robotic crystallization trials, small rhomboidal shaped crystals were obtained using precipitants containing divalent metal salts, including calcium acetate, magnesium acetate and magnesium formate. Crystallization conditions include conditions 18 and 46 (Crystal Screen HR2–110, Hampton Research), condition 20 (PEG/Ion Screen, HR2–126, Hampton Research) and condition 55 (Index screen HR2–144, Hampton Research). Larger crystals of rhombus or trapezoid shape were obtained over 2–5 days by mixing a volume of 1 μl of NS5 (6–895) at 4–6 mg/ml with 1 μl of precipitation solution (0.2 M calcium acetate or magnesium acetate, 0.1 M sodium cacodylate, pH = 6.4, 10–20% (w/v) PEG 8000). Crystals for the GTP complex were obtained by either co-crystallization of NS5 at a concentration of 6 mg/ml (~60 μM), with 0.4 mM GTP using a slightly different precipitating solution (0.1 M sodium cacodylate (pH 6.4), 0.2 M magnesium acetate or 0.2 M calcium acetate and 14% (w/v) polyethylene glycol 8000) at 18°C, or soaking of apo NS5 crystal in the precipitating solution supplemented with 5 mM GTP overnight. Although these crystals grow in slightly different conditions, they are isomorphous. Prior to data collection, crystals were soaked for a few seconds in a cryoprotecting solution containing 20% (v/v) glycerol before being mounted and cooled to 100 K in a nitrogen gas stream (Oxford Cryosystems). Diffraction intensities were collected at the PXIII (X10SA) beamline of the Swiss Light Source, Paul Scherrer Institut, Villigen, Switzerland. Diffraction intensities for cocrystals with GTP were collected at the National Synchrotron Radiation Research Center, beamline 13B1 (Hsinchu, Taiwan). The best diffraction data were obtained for crystals of NS5 grown in the presence of calcium acetate. Integration, scaling, and merging of the intensities were carried out using programs MOSFLM and SCALA from the CCP4 suite [35]. The asymmetric unit contains one NS5 molecule with S-adenosyl-L-homocysteine (SAH) (copurified from *E*. *coli*) bound to the MTase domain. Crystal parameters and data collection statistics are summarized in Table 1.

**Table 1:** Data collection and refinement statistics.

	NS5	NS5: GTP
Resolution range (Å)	39.18–2.30 (2.39–2.30)*	29.88–2.40 (2.50–2.40)
Space group	P 2_1_2_1_2	P 2_1_2_1_2
Unit cell parameters a, b, c (Å) (°)	95.13, 151.45, 69.12	94.86, 150.97, 69.28
Total reflections	142,366 (12,688)	230,234 (26,360)
Unique reflections	41,247 (4,050)	39,648 (4,402)
Multiplicity	3.5 (3.1)	5.8 (6.0)
Completeness (%)	91.8 (86.8)	99.75 (99.95)
Mean I/sigma(I)	9.5 (2.6)	11.2 (3.5)
R-merge	0.077 (0.333)	0.115 (0.500)
R-work	0.191 (0.266)	0.188 (0.210)
R-free	0.246 (0.337)	0.237 (0.280)
Number of non-hydrogen atoms	7,168	7,332
macromolecules	6,859	6,875
ligands	32	51
water	277	406
Protein residues	854	854
RMS(bonds, Å)	0.004	0.004
RMS(angles,°)	0.80	0.83
Ramachandran plot (%)		
favored	96.45	96.34
allowed	3.31	3.31
outliers	0.24	0.35
Clash score	4.91	7.22
Average B-factor	39.30	42.00
macromolecules	39.30	42.10
ligands	45.00	60.50
solvent	37.40	38.40

*Statistics for the highest-resolution shell are shown in parentheses.

### Structure solution and refinement

The crystals have an estimated solvent content of 50.2% based on a Matthews coefficient (Vm) value of 2.47 [36]. The structure was solved by molecular replacement with the program PHASER [37] using the DENV3 MTase (Protein Data Bank [PDB] accession code 3P97) [38] and DENV3 RdRp (PDB accession code 2J7U) [13] as search probes. Refinement cycles carried out using REFMAC5 [39] and PHENIX [40] were interspersed with manual model rebuilding sessions using Coot [41]. TLS refinement was introduced in the last refinement steps. The quality of the structure was analyzed using Molprobity [42]. A summary of structure refinement statistics is given in Table 1. Solvent-accessible surfaces areas were calculated using CCP4 program AREAIMOL with a 1.4-Å radius sphere as a probe. Superimpositions of structures were carried out using the program LSQKAB from the CCP4 suite. Figures were prepared using the program Pymol [43]. In the crystal structure of NS5 with GTP bound to the MTase domain (Table 1), the overall conformation of NS5 is very similar to the NS5: SAH structure: The root mean square deviation (r.m.s.d.) is 0.17 Å for a total of 719 Cα atoms superimposed. Buried surface areas were calculated with the PISA server (www.ebi.ac.uk/pisa) using the following domains definition: MTase residues 6–262 in DENV3 and 6–265 in JEV; RdRp residues 273–883 in DENV3 and 276–896 in JEV (S2 Table) [44]. The relative reorientation between domains was assessed using the program DynDom [45]. The refined coordinates were deposited in the PDB under accession codes 4V0Q (NS5: SAH) and 4V0R (NS5: SAH: GTP).

### Hydrogen/Deuterium Exchange (HDX)

The buffer for the HDX reaction was of the same composition (20 mM Na-Hepes, pH 7.5, 300 mM NaCl, 5 mM DTT, 10% (v/v) glycerol) except that H_2_O was replaced with D_2_O (99.99%) and glycerol was omitted. Essentially the deuterium exchange reactions for NS5, or the individual MTase or RdRp domains was carried out by mixing a volume of 4 μl of the respective protein solutions (at a concentration of 18 μM), with 16 μl D_2_O or H_2_O buffer and incubated at 4°C for 0s, 30s, 60s, 600s, 1800s. The deuterium exchange (final D_2_O concentration 80%) for each sample at the various time points was quenched by the addition of 20 μl ice-cold solution consisting of 1 M guanidine hydrochloride and 1.5% (v/v) formic acid followed by rapid freezing in liquid nitrogen and storage at -80°C until HDX MS analysis.

### In-column pepsin digestion, LCMS and HDX data processing

For capillary-flow LC, buffer A was H_2_O containing 0.3% (v/v) formic acid. Buffer B was acetonitrile containing 0.3% (v/v) formic acid. Deuterium exchanged protein samples from above were then digested online by passing through an immobilized pepsin-coupled column (2.1 mm i.d. × 30 mm) (Invitrogen) at 16°C and were de-salted for 3 min on a home-packed C4 trap (0.75 mm i.d. × 10 mm, C4 beads purchased from Michrom) with buffer A (H_2_O containing 0.3% (v/v) formic acid) at a flow rate of 150 μl min^-1^ driven by the LC loading pump (Dionex 3000 RSLC). The peptide samples were separated further on a home-packed C4 column (0.3 mm i.d. × 50 mm, C4 beads purchased from Michrom) using a 20 min gradient (5% to 35%) in buffer B (acetonitrile containing 0.3% (v/v) formic acid), followed by washing with 90% buffer B for 3 min and equilibration with 1% buffer B for 5 min) at a flow rate of 15 μl min^-1^ driven by LC NC pumps. MS raw data were acquired in the range of m/z 300–2000 for 30 min in positive mode on a LTQ-Orbitrap mass spectrometer (Thermo Fisher Scientific) equipped with an ESI source (capillary temperature 275°C and spray voltage of 5 kV). All the HDX separations were performed at 0°C except for on-line pepsin digestion that was carried out at 16°C. Blank injections were made between runs to remove carry-over peptides. At least three independent deuterium exchange experiments were carried out for each time point. All HDX data were normalized to 100% D_2_O content, corrected for an estimated average deuterium recovery of 70%, and analyzed by the software HD Desktop [46]. Initial peptic peptide identifications were performed with the same HDX set up as described above. 4 μl of protein sample (20 μM) was injected into the HDX MS system. Product ion (MS/MS) spectra were acquired in linear ion trap LTQ with eight most abundant ions selected in the precursor (MS) scan with a 7.5 sec exclusion time. MS and tandem MS files were extracted and searched by using the Global proteome machine (http://www.thegpm.org) for high-confident peptide identification.

### Generation of DENV4 NS5 mutant replicons

The K95A, Y119A, R263A (R262 in DENV3), E268A (E267 in DENV3), E270A (E269 in DENV3), and R353A (R352 in DENV3) mutants in the DENV4 NS5 sequence (GenBank accession number AF326825) were engineered into the subclone, pACYC-DENV4-F shuttle, using the QuikChange II XL site-directed mutagenesis kit according to the manufacturer’s protocol (Stratagene). This plasmid harbours nucleotides 7564–10653 (from NS3–3’UTR) from the DENV4, MY01–22713 strain, linked at the 3’ end to the Hepatitis D virus ribozyme (HDENVr) sequence [47]. Following sequence verification, the plasmids were digested with *NotI* and *KpnI* and inserted with a PCR product comprising the sequence spanning nucleotides 1–7563 under the control of the upstream T7 promoter in which the region from nucleotides 217–2291 in this cDNA had been replaced by renilla luciferase and foot-and-mouth disease virus 2A protease cDNAs [38]. A list of primers used for cloning and mutagenesis is available in S1A Table is S1 Text.

### *In vitro* transcription, RNA transfection, and renilla luciferase (RenLUC) measurements

After linearization of the corresponding replicon cDNA plasmids with XhoI, *in vitro* transcription (IVT) was performed using a T7 mMESSAGE mMACHINE kit according to the manufacturer’s protocol (Ambion; Austin, Texas, USA). IVT RNAs (10 μg) were electroporated into 8×10^6^ BHK-21 cells, after which the cells were re-suspended in a volume of 25 ml of DMEM with 10% FBS. Cell suspensions of 0.5 ml per well were seeded into 12-well plate; and the cells were assayed for luciferase activities at 4, 24, 48, 72, and 96 h post-transfection. Duplicate wells were seeded for each data point. Luciferase assays were performed using a RenLUC assay system following the manufacturer’s protocol (Promega, WI, USA).

### Cloning and protein expression of DENV4 WT and mutant NS5 proteins

NS5 (serotype DENV4) WT and mutant cDNAs were amplified by PCR from the respective WT and mutant pACYC-DENV4-F shuttle plasmids and cloned into the pET28a vector (Stratagene) using the *NheI* and *XhoI* sites. Protein expression and heat stability analyses by thermo-fluoresence were performed as described previously [32].

### DENV4 NS5 methyl-transferase *in vitro* assays

N-7 and 2’-*O* MTase assays were performed as described [38,48]. In general, the N7-MTase reaction comprised 25 nM protein, 240 nM biotinylated GpppA-DENV nt 1–110 *in vitro* transcribed RNA, 320 nM [^3^H-methyl]-SAM in 50 mM Tris-HCl, pH 7.5, 20 mM NaCl, and 0.05% (v/v) CHAPS. The 2’-*O* MTase reaction comprised 25 nM protein, 40 nM GpppA-7mer RNA (Trilink), 320 nM [^3^H-methyl]-SAM in 50 mM Tris-HCl, pH 7.5, 10 mM KCl, 2 mM MgCl_2_, and 0.05% CHAPS. Buffer, RNA substrate, and enzyme were first mixed in a single well in a 96-well half- area, white opaque plates (Corning Costar, Acton, MA), and the reaction was initiated by addition of [^3^H-methyl]-SAM. The N-7 and 2’-*O* reactions were incubated at RT for 15 min and 1 hr, respectively. The reaction was stopped with 25 μl of 2× stop solution (100 mM Tris/HCl, pH 7, 100 mM EDTA, 600 mM NaCl, 4 mg/ml streptavidin—SPA beads and 62.5 μM cold AdoMet) and shaken for 20 min at 750 rpm at room temperature followed by centrifugation for 2 mins at 1200 rpm. The plate was read in a Trilux microbeta counter (PerkinElmer, Boston, MA) with a counting time of 1 min per well. All data points were collected in duplicate wells.

### DENV4 NS5 polymerase *in vitro* assays

The *de novo* initiation/elongation assay was described [28,49]. Briefly, the reaction comprised 100 nM DENV4 NS5, 100 nM in vitro transcribed DENV4 5´UTR-3´UTR RNA, 20 μM ATP, 20 μM GTP, 20 μM UTP, 5 μM Atto-CTP (Trilink Biotechnologies), in a volume of 15 μl of the assay buffer comprising 50 mM Tris-HCl, pH 7.5, 10 mM KCl, 1 mM MgCl_2_, 0.3 mM MnCl_2_, 0.001% Triton X-100 and 10 μM cysteine. The elongation assay reaction comprised 100 nM IVT 244 nt heteropolymeric RNA template, annealed with four primers (C1 primer 3’-AGTCAGTCAGTCAGTGT-biotin-5’, A1 primer 3’-GTCAGTCAGTCAGTCTC-biotin-5’, G1 primer 3’-TCAGTCAGTCAGTCACA-biotin-5’, T1 primer 3’-CAGTCAGTCAGTCAGAG-biotin-5’) [50], 2 μM ATP, 2 μM GTP, 2 μM UTP, 0.5 μM Atto-CTP, and 100 nM of DENV4 NS5 in 15 μl in assay buffer comprising 50 mM Tris-HCl at pH 7.5, 10 mM KCl, 0.5 mM MnCl_2_, 0.01% Triton X-100, and 10 μM cysteine. RNA was separately pre-annealed to the four different sets of primers at a ratio of 1:2 (w / w) by heating at 95°C for 3 min, and cooled to RT before mixing and used for the assay. All reactions were allowed to proceed for up to 3 hrs at RT. At the indicated time-points, 10 μl of 2.5× STOP buffer (200 mM NaCl, 25 mM MgCl_2_, 1.5 M DEA, pH 10; Promega) with 25 nM calf intestinal alkaline phosphatase (CIP; New England Biolabs) was added to the wells to terminate the reactions. The plate was shaken and centrifuged briefly at 1200 rpm, followed by incubation at RT for 60 min and the released AttoPhos was monitored by reading on a Tecan Saffire II microplate reader at excitation_max_ and emission_max_ wavelengths 422 nm and 566 nm respectively. All data points were collected in triplicate wells in 384-well black opaque plates (Corning).

### Construction of mutant DENV2 full-length infectious clone

Mutations of K95A, Y119A, E268A (E267 in DENV3) and R353A (R352 in DENV3) were constructed into full-length DENV2 cDNA clone (GenBank accession EU081177.1) using the subclone pWSK29 D2 fragment 3 as described [17]. Briefly, mutations were generated using the QuikChange II XL site-directed mutagenesis kit (Stratagene) according to the manufacturer’s protocol. Primers used for mutagenesis are listed in S1D Table in S1 Text. Fragment 3 bearing the mutation was excised from the plasmid by *XbaI* and *SacI*, and inserted into subclone pWSK2 D2 fragment 1+2 that was similarly cut with *XhaI* and *SacI*.

### *In vitro* transcription and replication profiling of mutants

The genome-length wild-type DENV2 infectious clone plasmid pWSK29 (GenBank accession EU081177.1) and the mutants were linearized with *SacI* and purified with phenol-chloroform. A quantity of ~2 μg linearized DNA was used for *in vitro* transcription with T7 mMESSAGE mMACHINE kit (Ambion). BHK-21 cells were trypsinized, washed twice with cold PBS and resuspended in Opti-MEM (Gibco) at a cell density of 1 × 10^7^ cells/ml. 10 μg of *in vitro* transcribed RNA (wild-type DENV2 and mutants) were mixed with 800 μl cell suspension in a pre-chilled 0.4 cm cuvette, and electroporated at settings of 850 V and 25μ F, 2 pulses at 3 seconds intervals. Electroporated cells were allowed to recover at room temperature for 10 minutes prior to resuspending in complete RPMI 1640 media. 3 × 10^5^ cells were then seeded into a 12-well plate and incubated at 37°C in the presence of 5% CO_2_. Media was changed to 2% FBS maintenance media after 6 hours post-transfection. Samples were harvested every 24 hours post-transfection until 120 hours. Supernatants were harvested and clarified for determination of virus titer by standard plaque assay on BHK-21 cells and extracellular viral RNA quantification by real time RT-PCR. Cells were then washed once with PBS before lysing with Trizol reagent (Invitrogen) for total intracellular viral RNA quantification.

### Real time RT-PCR and Immunofluorescence assay

For extracellular viral RNA quantification, viral RNA was extracted from the supernatant using QIAamp Viral RNA Mini Kit (Qiagen) according to the manufacturer’s instructions. Real time RT-PCR was carried out in Bio-Rad Real time thermal cycler CFX96 by the use of iTaq Universal SYBR Green One-Step kit (Bio-Rad) with primers (forward, 5’- CAGGCTATGGCACTGTCACGAT-3’; and reverse, 5’-CCATTTGCAGCAACACCATCTC-3’) targeting for DENV2 E region (adopted from [51]). A plasmid fragment containing genome sequences of the E region was used to establish a standard curve for quantification of viral genome copy number per ml of supernatant.

For cellular viral RNA quantification, total RNA was extracted using Trizol extraction method and 40 ng RNA was subjected to real time RT-PCR as described above. Absolute copies of the viral RNA genome was normalized to actin expression with β-actin primers (forward, 5’- TCACCACACACTGTGCCCATCTACGA-3’ and reverse, 5’-CAGGGGAACCGCTCATTGCCAATGG-3’); and reported as viral RNA genome copy per μg RNA.

IFA against dsRNA with anti-dsRNA mAb J2 (Scicons) and NS5 protein with anti-NS5 hAb 5R3 were performed as described [52]. Images were captured on an inverted fluorescence microscope (Olympus IX71, Center Valley, USA) at 20× magnification and image analysis was done with ImageJ software.

## Results

### A compact conformation of NS5 from DENV3 with an inter-domain interface dominated by electrostatic interactions

**Fig 1:**
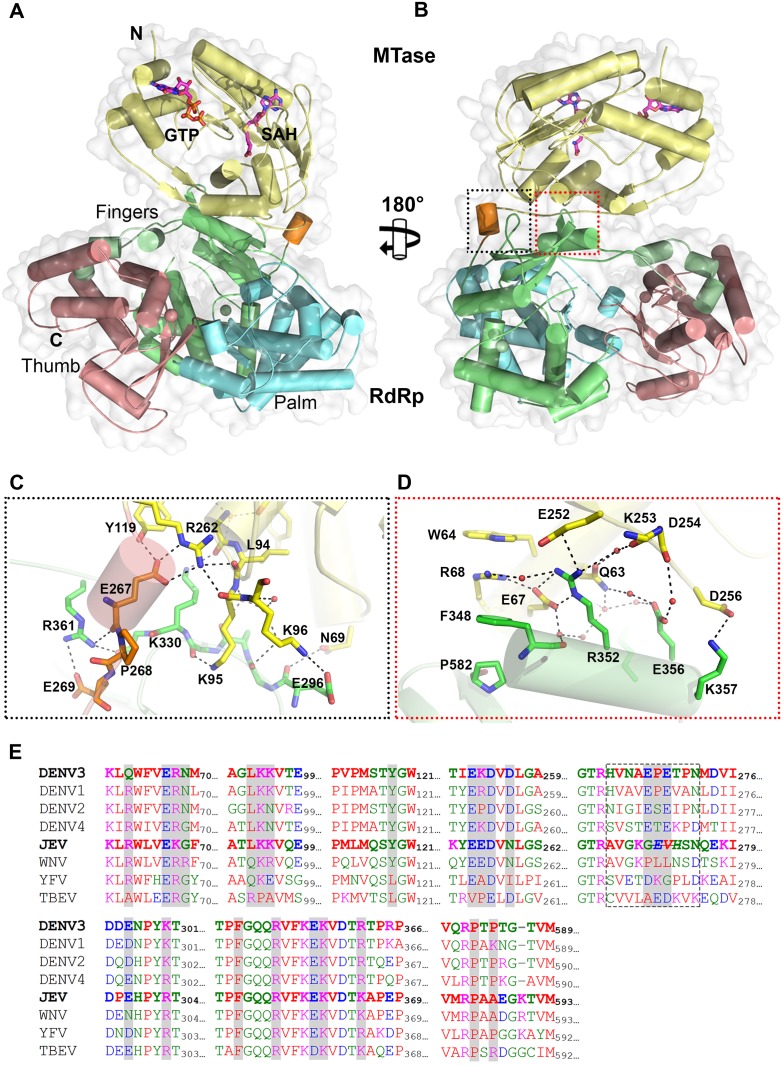
Crystal structure of DENV3 NS5. **(A)** Overall structure of the NS5 protein from DENV3 in cartoon representation viewing from the bottom of RdRp. MTase is in yellow, RdRp fingers in green, palm in blue, thumb in salmon. The linker helix (residues 263–267) between the two domains is in orange. GTP and co-factor SAH are shown as sticks and labelled. Zinc ions are shown as spheres. **(B)** View of the NS5 molecule from the top of RdRp, which is rotated by 180° around a vertical axis as in (A). Interface regions are boxed. **(C)** and **(D**) Close-up views of the interface between the MTase domain and RdRp domain as indicated in (B). Key residues for inter-domain interactions are shown as sticks and labeled. **(E)** Multiple sequence alignment of flavivirus NS5 proteins. Interface residues are highlighted in gray. The linker residues (263–272 in DENV3 NS5) are boxed. List of accession numbers for genes and proteins used for alignment: DENV3: gi|50347097|gb|AAT75224.1|; DENV1: gi|194338413|gb|ACF49259.1|; DENV2: gi|266813|sp|P29990.1|; DENV4: gi|425895219|gb|AFY10034.1|; JEV: gi|4416167|gb|AAD20233.1|; WNV: gi|607369775|gb|AHW48802.1|; YFV: gi|27735297|ref|NP_776009.1|; TBEV: gi|1709707|sp|Q01299.1|.

Overall, the polypeptide chain of NS5 is well defined in the electron density map, including for inter-domain residues 262–273 (Fig. 1 and Table 1). The NS5 protein from DENV3 adopts a compact shape with overall dimensions 87 Å × 72 Å × 55 Å (Fig. 1A and B). The MTase domain is located above the fingers subdomain of the RdRp (Fig. 1A), such that the GTP binding pocket, the K_61_D_146_K_180_E_216_ catalytic tetrad and the SAH binding pockets of the MTase domain are positioned away from the inter-domain interface. In this conformation, the tunnels of the RdRp domain that permit the entry of NTPs and RNA template remain accessible. The MTase-RdRp inter-domain interface is comprised of two contact areas involving linker residues, the fingers subdomain from the RdRp and three segments (residues 63–69, 95–96 and 252–256) from the MTase domain (Fig. 1). The network of interactions stabilizing the interface mainly comprises charged residues (Fig. 1; S2 Table in S1 Text). In the first cluster, linker residues interact with both the RdRp and MTase domains. The side chain of E267 hydrogen bonds with residues Y119 and R262 from the MTase domain, while E269 forms a salt bridge with the guanidinium group of R361 from the RdRp domain (Fig. 1C). Several polar interactions are also formed between residues K95-K96 from the MTase domain and E296-K300 from the RdRp domain (Fig. 1C; S2 Table in S1 Text). The second cluster of inter-domain interactions is centered at helix α5 (residues F348-K357), which belongs to the evolutionary conserved bNLS motif of the RdRp domain, (Fig. 1B and D). Projecting from helix α5, the guanidinium side chain of R352 plays a central role via the formation of numerous contacts including electrostatic interactions with E67, E252 and Q63. Another salt bridge is formed between K357 and D256 (Fig. 1D). In addition, several water molecules trapped at the interface mediate H-bond interactions between residues projecting from both domains (Fig. 1C and D). In contrast to multiple non-polar contacts established between the MTase and RdRp domains of NS5 from JEV, the only hydrophobic contacts between the two domains of NS5 from DENV3 involve stacking interactions between W64, R68, F348 and P582 (Fig. 1D).

**Fig 2:**
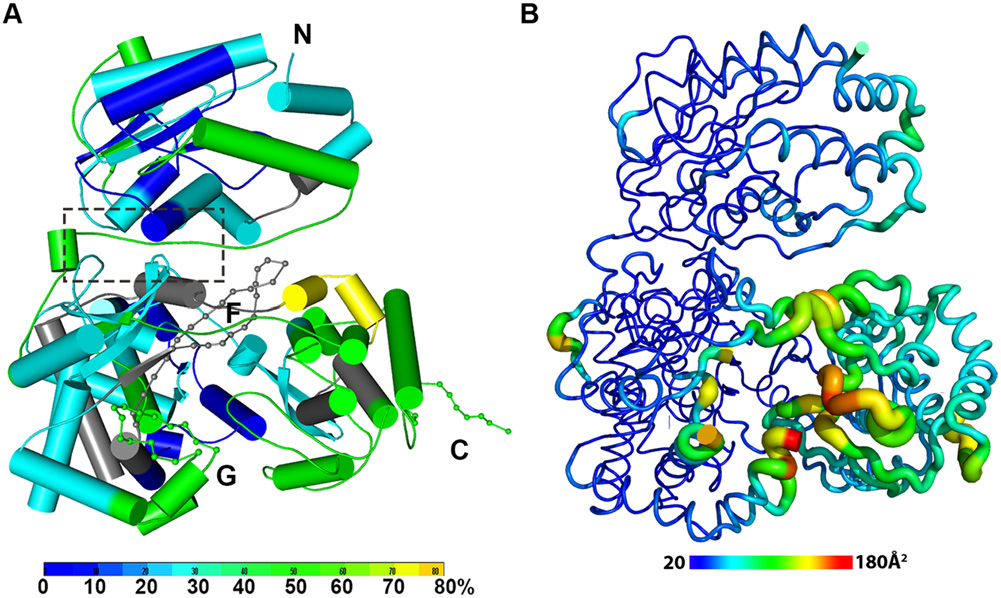
The dynamics of NS5 in solution probed by HDX-MS. **(A)** Heat map data overlaid onto a crystal structure for the DENV3 NS5. The color key indicates the extent of the deuterium uptake (%), in which blue means the lowest deuterium uptake. Peptides in gray are regions where information of deuterium uptake is missing. F: motif F; G: motif G. **(B)** Putty cartoon view of temperature factor variation on the crystal structure of DENV3 NS5, colored from low to high values (20~180 Å^2^ as blue to red).

Despite an extensive inter-domain interface (Fig. 2), the MTase and RdRp domains of NS5 display only minimal differences at surface exposed regions compared to the individual enzymatic domains (S3 Fig in S1 Text). Superimposition with the NS5-MTase domain structures (PDB codes 3P97 and 1L9K) returns RMSD values of 0.42 Å and 0.40 Å for 221 Cα and 219 Cα atoms respectively [5,38]. In the MTase domain, the co-purified (SAH) molecule resides in the cofactor-binding pocket and is stabilized by a network of hydrogen bonds and van der Waals contacts (Fig. 1; S1 Fig in S1 Text) essentially identical to those reported earlier [5,53,54]. GTP is stabilized by base stacking with the aromatic ring of F25 and electrostatic interactions with residues L17, N18, and L20 of the MTase domain (S1 Fig in S1 Text) [5,54]. Likewise, superimposition with the DENV3 NS5-RdRp (PDB codes 2J7U and 4A11) yields RMSD values of 0.47 Å and 0.43Å for 492 and 495 Cα atoms respectively [13,32]. Like in the isolated RdRp domain structure reported earlier [13], however, two segments of the polypeptide chain are disordered in the present NS5 structure: residues 406–417 that harbor motif G, which regulate access of the ssRNA substrate to the template tunnel [13] and residues 455–468 which contain motif F. The latter were proposed to bind Stem Loop A, the viral promoter, prior to viral RNA replication [55]. Two Zinc ions bound to the RdRp domain are also observed as reported earlier [12,13].

### Conformational dynamics of NS5 in solution

In order to determine whether the conformation of NS5 captured in the crystal state agrees with that in solution and also to better understand the dynamics of NS5, we performed Hydrogen/Deuterium Exchange Mass Spectrometry (HDX-MS) experiments using NS5, NS5-MTase or NS5-RdRp (Fig. 2A; S2 Fig in S1 Text). HDX-MS results are influenced by protein exposure to solvent and stability in solution, making this technique suitable to study solution-state protein dynamics [17,56–59]. The HDX-MS heat maps were overlaid onto the three respective crystal structures, with a color code indicating the extent of deuterium exchange with the protein backbone amide hydrogen atoms. The missing residues 406–417, 455–468, and 884–891 were modeled based on the homology to JEV NS5 structure. The corresponding HDX-MS profiles of peptic peptides across NS5 are listed in S2A Fig in S1 Text. The interface peptides from the MTase (residues 65–77 and 95–118) and the RdRp (residues 291–301 and 581–587) domains of NS5 display comparable deuterium exchange (Fig. 2A, boxed area; S2E and S2F Fig in S1 Text). However, the extent of deuterium exchange of these peptides (~ 20–30%) is not as low as the domain core region (~ 10% in blue), suggesting that the interface is solvent accessible as indicated by the crystal structure (Fig. 2A; S2 Fig in S1 Text).

Interestingly the HDX-MS also reveals the local protein dynamics of NS5. Peptides from the thumb subdomain (I735-L748, S781-M809, V812-L850, and L853-S895) and the middle finger (K311-M340) of the RdRp domain display higher deuterium-exchange (Fig. 2A; S2 Fig in S1 Text) in NS5 compared with the isolated NS5-RdRp domain. This suggests that in the context of the full length NS5 protein, the thumb subdomain of RdRp is more dynamic in solution than its fingers and palm subdomains. Remarkably the dynamics measurements with HDX-MS correlated very well with the distribution of crystallographic temperature factors observed for the RdRp domain of NS5, where higher temperature factors for the thumb subdomain than the fingers and palm subdomains are observed (Fig. 2B). Likewise, residues containing motifs F and G that are disordered in the crystal structure of NS5, also display a higher degree of amide hydrogen exchanges in HDX-MS (Fig. 2A; S2 Fig in S1 Text) [13,32]. It is worth noting that the conformation adopted by motifs F and G in the crystal structure of the isolated RdRp domain is different in the NS5 protein from JEV (S4 Fig in S1 Text). Overall, the HDX-MS experiment indicates that the conformation adopted by DENV3 NS5 in the crystal structure is also accessible in solution but that other conformations are possible.

### Structure of the linker as the determinant of NS5 inter-domain flexibility

**Fig 3:**
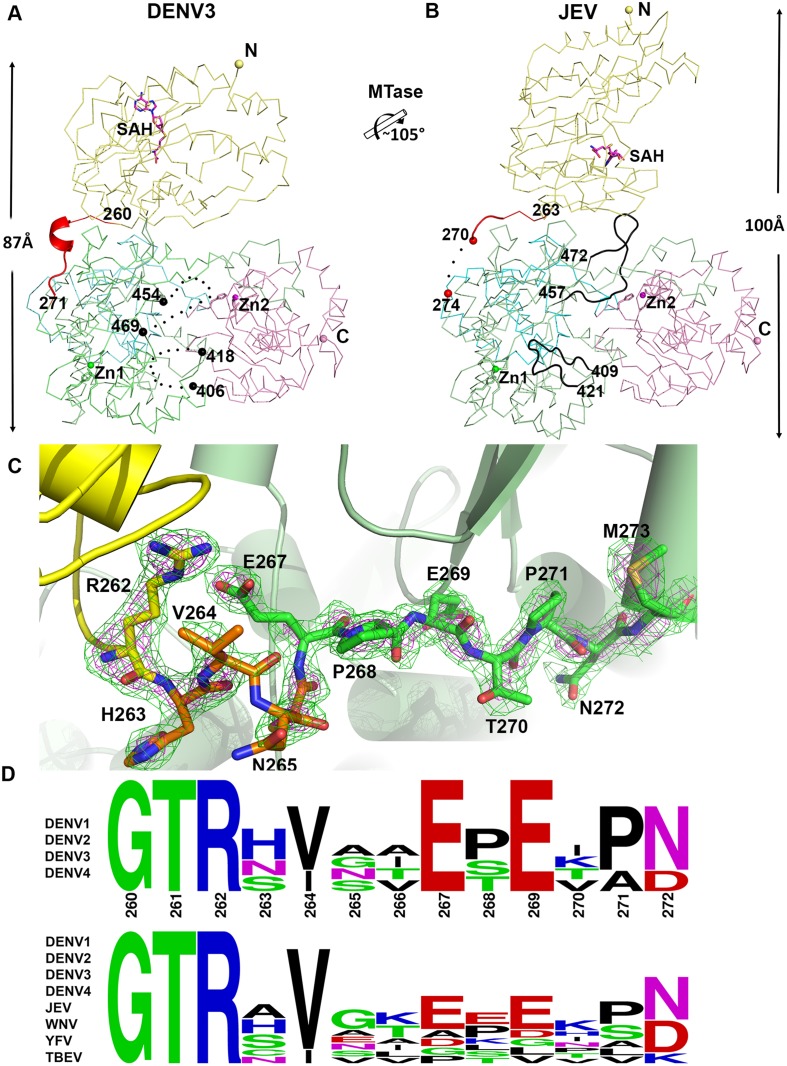
Comparison of conformations between DENV3 NS5 and JEV NS5 structures. **(A)** and **(B)** Side-by-side view of the DENV3 NS5 and the JEV NS5 structures in thin ribbon style. Same color scheme as in Fig. 1: MTase is in yellow, RdRp fingers in green, palm in blue, thumb in salmon. The linker regions (260–271 in DENV3 NS5; 263–274 in JEV NS5) are shown as cartoon in red. Missing residues (407–417 and 455–468 in DENV3 NS5; 271–273 in JEV NS5) are indicated as dots. The rotation axis that relates both MTase domains is indicated with the corresponding angle. **(C)** The simulated annealing mF_obs_-DF_calc_ omit maps are in green, contoured at 3σ and in magenta, contoured at 5σ. **(D)** Sequence conservation of the linker region of DENV 1–4 and representative flaviviruses. Figure is generated with WebLogo [71].

Despite a similar molecular shape maintained by intra-molecular interactions between the MTase and fingers subdomain of the RdRp, both the relative orientation and the interfaces between domains differ between the NS5 proteins from JEV and DENV3 (Fig. 3) [31]. Following superimposition of their RdRp domains, an additional rotation of 105° of their MTase domain is needed to bring them to coincidence (Fig. 3; S1 Movie). The inter-domain linker regions also differ markedly between the two proteins: In DENV3 NS5, the linker residues are well ordered (Fig. 3A and C), while in the JEV NS5 structure, residues A266-G270 adopt an extended conformation and residues E271-V272-H273 are disordered (Fig. 3B; S4 Fig in S1 Text) [31]. Since the linker residues are well ordered with clear electron density in the present DENV3 NS5 crystal structure (Fig. 3C), a precise definition of the structural determinants contributing to inter-domain flexibility is now possible. Earlier crystal structures of NS5 individual domains suggested that the evolutionarily conserved R262, whose guanidinium group hydrogen bonds with the main chain carbonyl oxygen of V97, defines the C terminus of the MTase domain (Fig. 1C; S3 Fig in S1 Text) [1,28,32,57,60]. Residues H263-V264-N265-A266, which are located after the C-terminal end of the MTase domain, are the least evolutionarily conserved in the interface region (Fig. 1E and 3D). Interestingly, these residues fold into a short 3_10_-helix giving the polypeptide chain a compact conformation and allowing the two domains of NS5 to form a large interface leading to its observed globular shape (Figs. 1 and 2). Conversely, the segment E267-N272 forms extensive interactions with both the MTase and RdRp domains (Fig. 1C and 3C). The side chain of E267 projects towards the MTase domain forming hydrogen bonds with R262 and Y119, while the side chain of E269 projects towards the RdRp domain and interacts with R361 and K595. The two acidic residues E267 and E269 are highly conserved among all four dengue serotypes, but not in other flaviviruses (Fig. 1E and 3D). Likewise, T270 and N272 establish several polar contacts with the RdRp domain (Fig. 1C). To further delineate the boundaries of the RdRp domain of NS5, we observe that residues 268–271 adopt the same conformation in both the DENV3 NS5-RdRp and in the present NS5 structures (S3 Fig in S1 Text) [32]. Coincidentally, residues 268–272 enhance the thermostability of the truncated NS5-RdRp and also its polymerase activity [32] suggesting that they may be considered as an integral part of the RdRp domain of NS5 (Fig. 3D; S3 Fig in S1 Text). We conclude that DENV NS5 contains a short four-amino-acids long inter-domain linker (residues 263–266). Like a swivel, it is conceivable that structural transitions at this short linker—for instance a conversion from a helix to more extended conformations—are accompanied by important changes in the relative orientation between the two domains of NS5 [61].

### Comparison of inter-domain interface

**Fig 4:**
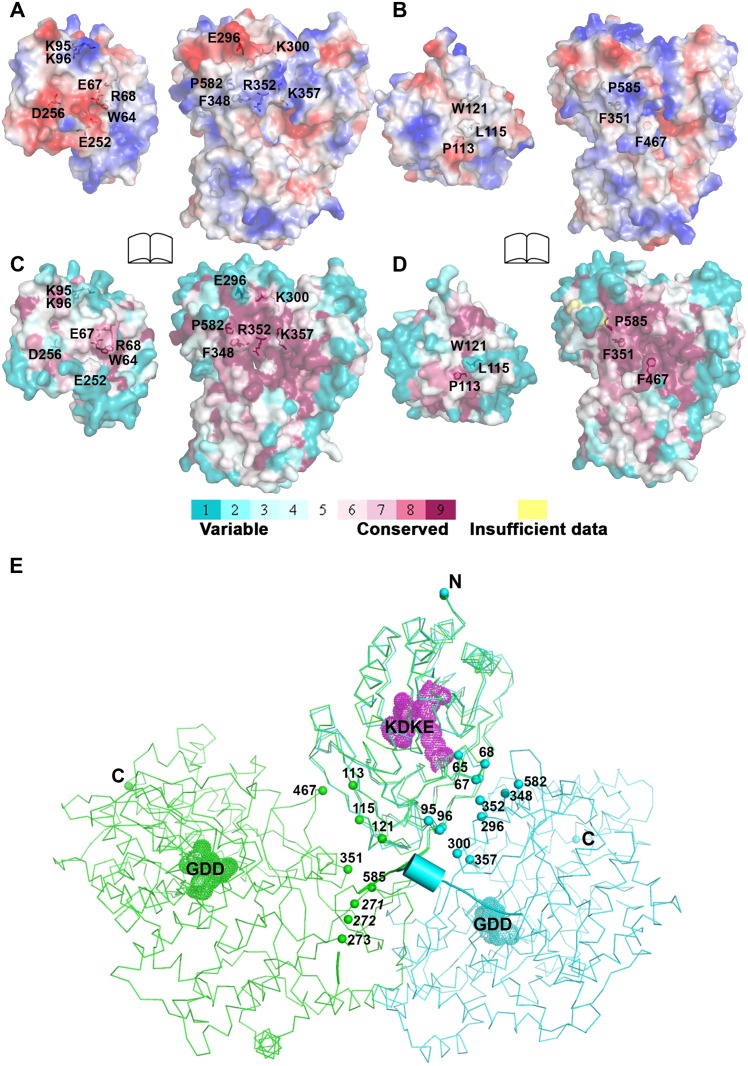
Comparison of interfaces between DENV3 and JEV NS5. RdRp domains of the two structures have been displayed in the same orientation and the buried surfaces (indicated by the labeled interface residues) from the RdRp domains are relatively overlapping between the two NS5 structures. **(A)** and **(B)** Analysis of the electrostatic properties of the domain interfaces of the two NS5 structures. Positive surface charge is highlighted in red, negative charge in blue. **(C)** and **(D)** Analysis of the evolutionary conservation of the domain interfaces of the two NS5 structures. The color key indicates the degree of conservation, with cyan means highly variable and purple means highly conserved. **(E)** The relative orientations of the RdRp domain relative to the MTase domain in two structures in thin ribbon style. DENV3 NS5 is in blue, JEV NS5 is in green. “KDKE” highlights the MTase active site shown as mesh in purple and “GDD” for the RdRp active site shown as mesh. Residues at interface are highlighted and shown as dots. Linker (262–272) in DENV3 NS5 is shown as cartoon.

The total buried interdomain surface area calculated for DENV3 NS5 is 1502 Å^2^ which is only slightly larger than that for JEV NS5 (1464 Å^2^) (Domain definition: MTase residues 6–262 in DENV3 and 6–265 in JEV; RdRp residues 273–883 in DENV3 and 276–896 in JEV) (S2A Table in S1 Text) (http://www.ebi.ac.uk/pdbe/pisa/) [44]. These values suggest that both structures are relatively stable in solution [62]. This is consistent with the rather compact shapes adopted by NS5 with maximum molecular dimensions of approximately 87 Å (DENV3) and 100 Å (JEV) (Fig. 3A and B). Interestingly, major inter-domain interactions in DENV3 NS5 are of polar nature, occurring both directly between charged side-chains and through water-mediated contacts (Fig. 4A; S2B Table in S1 Text). In contrast, the inter-domain interactions in JEV NS5 are predominantly hydrophobic, including residues P113, L115, W121 from MTase and F467 (F464 in DENV3), F351(F348 in DENV3) and P585 (P582 in DENV3) from the RdRp domain (Fig. 4B; S2B Table in S1 Text) [31]. An analysis of interface residues reveals that residues in both interfaces were highly conserved during evolution (Fig. 4C and D) [63]. When superposing MTase domains of the two NS5 structures, the relative positions of the catalytic centers of the MTase domain (KDKE catalytic tetrad) and the RdRp domain (GDD, motif C) are far apart (Fig. 4E). It is thus evident that both DENV and JEV RdRp domains used the finger subdomains to interact with their respective MTase domains from two non-overlapping sides, thus establishing unique interfaces for DENV or JEV (Fig. 4).

### Differential effects of interface mutations on *in vitro* enzymatic activities of NS5

To examine the functional importance of individual residues within the two clusters of interactions at the DENV NS5 inter-domain interface (Fig. 1C and D), we introduced single alanine mutations at residues K95, Y119, R263 (R262 in DENV3), and R353 (R352 in DENV3) into the DENV4 NS5 protein and compared their polymerase activities against the WT protein (Table 2). Note also that the residues selected for mutagenesis are conserved across the four DENV serotypes but not across other flaviviruses, except for R263 (R262 in DENV3) and R353 (R352 in DENV3) (Fig. 1E). All purified mutant NS5 proteins retained similar stability as WT as determined by a thermofluor assay, suggesting a native fold (Table 2). The RdRp activity of the mutant proteins compared to WT enzyme was assessed by both the *de novo* initiation/elongation assay and the elongation assay as reported [32]. The former uses the viral UTR sequence as template to start a new RNA chain while the latter employs an heteropolymeric RNA template annealed with four primers, which is extended during the course of the assay [28,49].

**Table 2:** Enzymatic activities and thermo-stabilities of DENV4 WT and mutant NS5 proteins.

% NS5 activity	*De novo* initiation/elongation			Elongation			N-7 MTase	2’-*O* MTase	*Thermo-fluorescence*
Time (hr)	1	2	3	1	2	3	0.25	1	Tm (°C)
WT	100	100	100	100	100	100	100	100	37
K95A	189.9 ± 4.0	198.4 ± 9.5	171.9 ± 3.7	194.0± 3.9	200.1 ± 1.0	182.3 ± 13.1	85.6 ± 15.0	82.4 ± 1.0	37
Y119A	149.5 ± 11.4	144.3 ± 22.1	149.9 ± 12.1	113.0 ± 3.3	110.2 ± 1.1	114.5 ± 0.5	46.3 ± 0.8	8.3 ± 0.4	38
R263A	151.3 ± 2.5	142.8 ± 4.9	135.7 ± 5.0	103.9 ± 4.8	105.0 ± 0.5	112.4 ± 0.8	4.0 ± 0.3	3.2 ± 0.3	37
E268A	132.8 ± 12.1*	123.9 ± 1.3*	146.4 ± 1.7*	92.7 ± 2.5	87.6 ± 2.8	98.3 ± 3.2	71.9 ± 2.6	70.4 ± 2.5	37
E270A	47.2 ± 6.4*	47.4 ± 5.6*	54.6 ± 10.2*	71.7 ±2.4	70.1 ± 1.8	77.9 ± 1.5	74.1 ± 10.2	65.1 ± 0.4	37
R353A	107.0 ± 3.1	92.0 ± 3.4	94.3 ± 2.5	193.1 ± 2.9	198.1 ± 25.1	206.4 ± 15.0	106.3 ± 6.7	97.7 ± 4.4	38

Polymerase activities DENV4 WT and mutant NS5 (full length) proteins measured in *de novo* initiation and elongation FAPA assays. Results shown are the average percentage activity compared against DENV4 WT NS5 protein derived from average relative fluorescence units (RFU) obtained for each protein from one experiment. All data points were performed in duplicate wells. N7 and 2’-O MTase of DENV4 WT and mutant NS5 proteins measured in SPA assays. Results shown are the average percentage activity compared against DENV4 WT NS5 protein derived from average corrected counts per minute (CCPM) obtained for each protein. Thermo-stability was assessed using the thermo-denaturation assay.

Footnote:

* Data source [32]. The effect of alanine mutations of E268 (E267 in DENV3), and E270(E269 in DENV3), on *de novo* initiation/elongation activities of polymerase were previously studied in Lim et al., 2013 and is included here for comparison.

Overall, with the exception of E270A (E269 in DENV3) that exhibits about 30–60% reduction in both polymerase activity, none of the NS5 mutants exhibit significant impairment in their *de novo* initiation or elongation activities. Mutants Y119A, R263A (R262 in DENV3), and E268A (E267 in DENV3) display a 24–50% increase in *de novo* initiation/elongation activities compared to WT NS5 (Table 2). The alanine mutation at K95 results in the most active RdRp so far with an almost 2 fold increase in both the *de novo* initiation/elongation and elongation activities, compared to WT. Residue R353 (R352 in DENV3) is located at the second interface cluster and belongs to a 20-amino-acids sequence conserved in all flavivirus NS5 proteins [15] and, as mentioned earlier, it forms part of the bNLS. The *de novo* initiation/elongation activity is 2-fold higher (similar to K95A) but the primer elongation activity is similar to WT (Table 2).

Next, we investigated the effect of the mutations on the N7 and 2’-*O* MTase activities of NS5 (Table 2). Mutation of residues Y119 and R263 (R262 in DENV3) to alanine strongly impacted both N7 and 2’-*O* MTase activities. Mutant Y119A exhibited less than 50% N7 activity compared to WT protein and minimal 2’-*O* activity. Both enzymatic activities were almost abolished in R263A (R262 in DENV3), the last residue of the MTase domain. On the other hand, E268A (E267 in DENV3) and E270A (E269 in DENV3)—mutants C-terminal to the inter-domain linker, retained 65–74% of both MTase activities, suggesting minor allosteric effects from the RdRp domain transmitted via the inter-domain interface. In contrast to the elevated polymerase activities, the K95A and R353A (R352 in DENV3) mutants that disrupt the transverse polar contacts appears to have little impact on the MTase activities (Table 2).

### Essential role of the inter-domain interface in regulating virus replication

**Fig 5:**
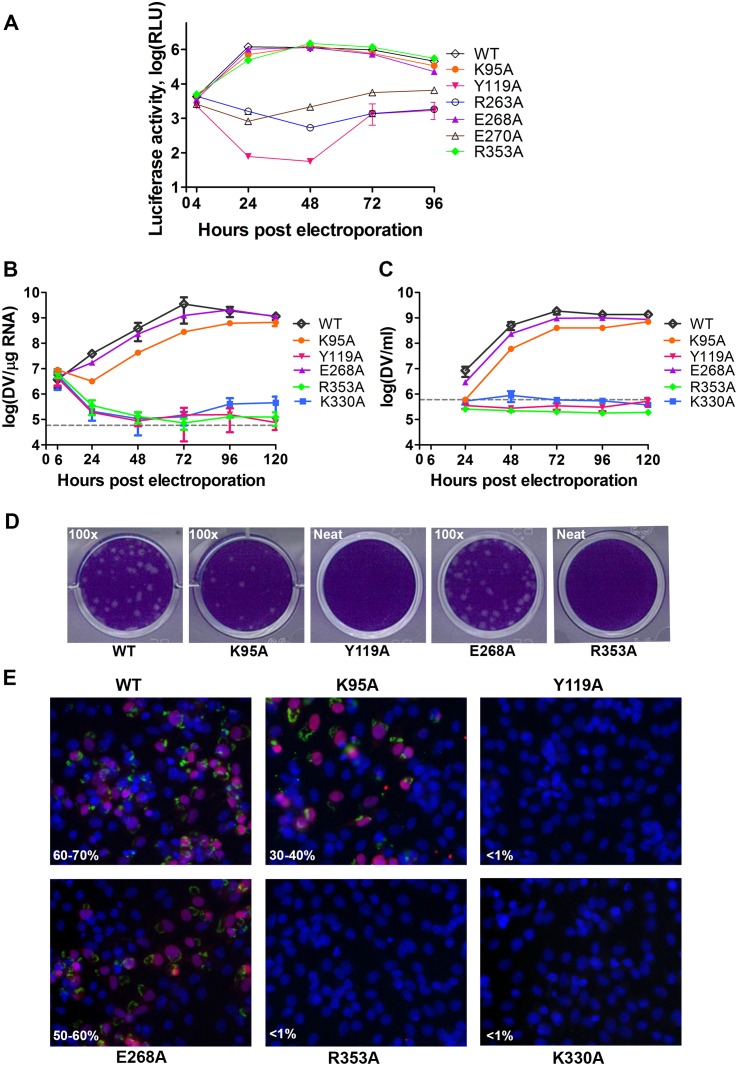
Replication profiles of NS5 interface mutants. **(A)** Renilla luciferase activities of DENV4 WT and mutant replicons. Equal amounts of replicon RNA (WT or mutants) were electroporated into BHK-21 cells. At the indicated time points, the transfected cells were lysed and assayed for luciferase activities. The y axis shows the log10 value of Renilla luciferase activity (RLU). Each data point is the average for three replicates, and error bars show the standard deviations. **(B)** 10μg *in vitro* transcribed infectious clone RNA was electroporated into BHK-21 cells and viral replication was monitored over a course of 5 days. Intracellular viral RNA replication as detected by qRT-PCR. The grey dotted line represents the background detection of uninfected cells. **(C)** Extracellular viral RNA in the supernatants detected by qRT-PCR. The grey dotted line denotes background signal of uninfected supernatant. **(D)** Plaque morphologies of WT and the mutants at 72 hours post electroporation. **(E)** IFA images showing dsRNA and NS5 co-staining and percentage infection of cells at 72 hours post electroporation.

**Fig 6:**
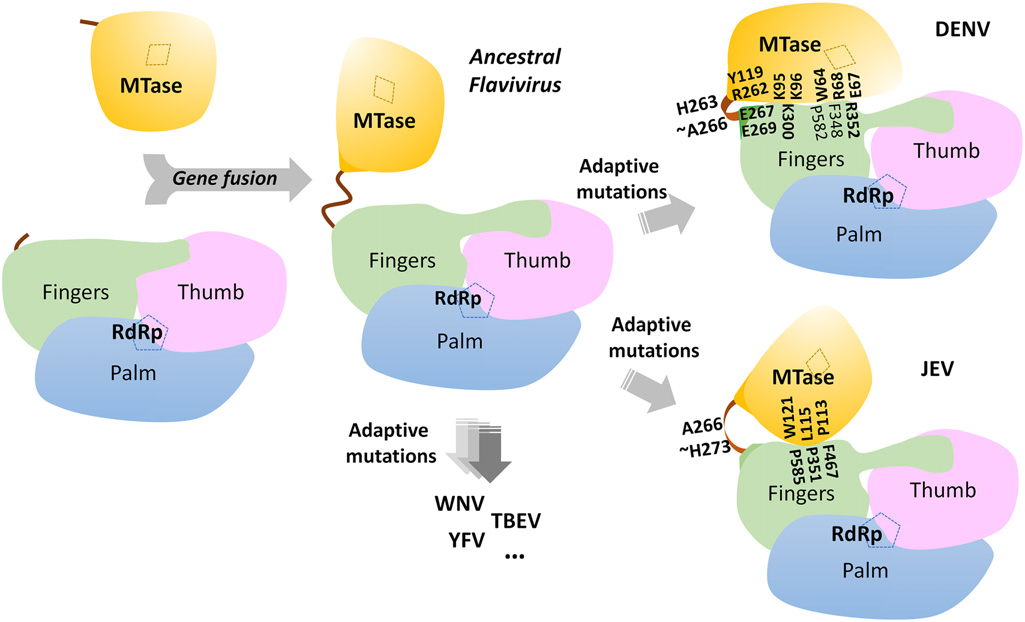
A schematic model for the divergent evolution of flaviviral NS5 proteins. The same color scheme as in Fig. 1 is used: MTase is in yellow, RdRp fingers in green, palm in blue, thumb in salmon. The linker region 3_10_ helix (residues 263–266) between the two domains is in orange. Active sites for MTase and RdRp are labelled with dotted tetragon and pentagon respectively. Linker residues and interface residues are labeled. A possible evolutionary pathway is presented: the MTase domain and RdRp domain originally existing as two separate proteins (left) became linked together to form the NS5 protein from an ancestral Flavivirus, possibly through gene fusion. This fusion promoted colocalization of both enzymatic activities and effectively increased the effective concentration of the proteins with respect to each other (middle panel). Following further (divergent) evolution, NS5 acquired different adaptive mutations and gave rise to the NS5 proteins now observed for various viruses, including DENV, JEV and possibly other flaviviruses) (right panel). Thus NS5 proteins from DENV and JEV may have different conformations and different allosteric mechanisms, in which the MTase and RdRp domain cross-talk to each other through unique interfaces specific to either DENV or JEV [72].

We next assessed whether residues involved in MTase-RdRp interactions are important for viral replication by introducing alanine mutations at positions K95, Y119, R263 (R262 in DENV3), E268 (E267 in DENV3), E270 (E269 in DENV3), and R353 (R352 in DENV3) in a DENV4 subgenomic RNA replicon, bearing a renilla luciferase reporter (Figs. 5A and 6) [32]. Both WT and mutant DENV4 replicon cDNAs were transcribed *in vitro*, electroporated into BHK-21 cells and luciferase activities were monitored at 4, 24, 48, 72 and 96 hr post-electroporation (Fig. 5A). The luciferase activity in cells harbouring WT DENV4 replicon showed a typical pattern, where a peak level is reached at approximately 24 to 48 hr post-electroporation (about 3320-fold above background levels), which steadily declined from 48–96 hr post-electroporation (Fig. 5A). Little or no luciferase activity was detected for DENV4 mutant replicons Y119A, R263A (R262 in DENV3), and E270A (E269 in DENV3), throughout the four days post-electroporation. Mutant replicons E268A (E267 in DENV3) and R353A (R352 in DENV3) exhibited significant luciferase activity that are almost comparable to WT at 48 hr point, although the mutants had some delay at the first 24 hrs (Fig. 5A).

To further validate the biological significance of the interface, four residues including K95, Y119, E268 (E267 in DENV3), and R353 (R352 in DENV3) were selected and mutated into alanine in the DENV2 infectious clone (Fig. 5B, C, and D). Defective mutant K330A, which has previously been reported, was included as a negative control [14,17]. E268A mutant (E267 in DENV3) displayed similar growth kinetics, extracellular viral RNA level and infectious virus recovery compared to WT. Y119A mutation did not yield any viable virus or increased intracellular RNA quantified by qRT-PCR. This is likely due to the defective MTase activity (Fig. 5; Table 2). The K95A mutant virus replicated much slower than WT with a 10-fold lower intracellular and extracellular RNA level at 72 hr post electroporation. No RNA replication or virus recovery was detected for mutant R353A (R352 in DENV3). Immuno-fluorescence analyses at 72 hr using anti-dsRNA and anti-NS5 antibodies confirmed the intracellular levels for WT and mutant viruses (Fig. 5E). Taken together, these data demonstrated: (i) most interestingly, conserved residues that form the major inter-domain polar contacts, K95 and R353 (R352 in DENV3), play critical but non-enzymatic roles in virus RNA replication and infectivity; (ii) alanine mutation of E268 (E267 in DENV3) have no impact on the enzymatic activities of NS5 nor on virus RNA replication and infectivity (iii) Within the MTase domain, Y119, is important for the MTase enzymatic activity of NS5 and is therefore essential for the virus replication.

## Discussion

### A well-ordered linker region defines the boundaries for MTase and RdRp domains of DENV NS5

The present 3D structure of DENV3 NS5 at 2.3 Å provides the first view of a flavivirus NS5 protein showing how its MTase and RdRp domains are physically connected through a fully resolved linker region (Fig. 1 and 3). Based on the present study and recent work [32], we conclude that DENV NS5 contains a four amino-acids long inter-domain linker (residues 263–266), which is able to fold into a short 3_10_-helix as observed in the present structure, but which can probably also adopt extended conformations (Fig. 6). In contrast, the JEV NS5 structure reveals a partially resolved linker region (266–275) which neither forms any secondary structure nor establishes interactions with either enzymatic domain (Fig. 3; S3 and S4 Fig in S1 Text) [31]. The low level of sequence conservation and the diverse conformations observed for the linker region strongly suggest conformational plasticity at the inter-domain interface for the flavivirus NS5 protein (Figs. 1 and 3D; S1 Movie). About 13 Å shorter in its largest dimension, DENV3 NS5 displays a more compact conformation than JEV NS5 (Fig. 3). This is reflected by the differences in the spatial arrangement of the two enzymatic domains (Fig. 4E). These different relative orientations are likely to result from different secondary structures adopted by the linker region that acts like a swivel, leading to the formation of different interfaces and enabling the two enzymatic domains to adopt various relative orientations during the transition from RNA synthesis to RNA capping (Figs. 3 and 6).

### The MTase and RdRp domains engage in cross talk through a unique electrostatic-rich interface specific to DENV

Remarkably, the interface in DENV3 NS5 is mostly polar with several salt bridges and fewer hydrophobic stacking interactions than that of JEV NS5, which is mainly of hydrophobic nature (Fig. 4A and B) [60]. The interface peptides from MTase domain and the fingers subdomain of RdRp displayed similar hydrogen deuterium exchange patterns, which suggest that the crystal structure of NS5 represents a predominant conformation in solution (Figs. 1 and 2A). However, given the polar nature of the interface interactions, conformational flexibility and heterogeneity are likely. An earlier SAXS study also suggested that the interaction between the MTase and RdRp domains may be heterogeneous: approximately 80% of DENV3 NS5 were found to adopt a relatively compact structure in which the two domains interact with each other whilst the remaining 20% of the population were in more extended conformations [29]. Therefore the two enzymatic domains of DENV NS5 may be able to associate and dissociate from each other with a relatively low energy barrier, consistent with a limited inter-domain buried surface area and the predominance of polar interactions. The prevalence of a more compact conformation in solution is consistent with our observation of a relatively short but structured four-residue-linker (residues 263–266 in DENV3 NS5) that restricts inter-domain movements. Furthermore, the solution HDX-MS is consistent with the temperature factor analysis of the DENV3 NS5 structure, indicating that the RdRp domain of DENV3 NS5 adopts a more flexible conformation especially in its thumb sub-domain (Fig. 2). These observations rationalize the contributions of a covalently tethered MTase domain to the RNA synthesis activities by RdRp [33]. In addition, the dynamic nature of the DENV NS5 protein may also facilitate the recruitment of other viral or host proteins. In agreement with this proposal, we have shown previously that K330 within the α3-helix of RdRp is critical for binding to the NS3 helicase. Mutation of K330 to alanine did not inhibit the *de novo* initiation and elongation activities in RdRp nor the 2’O and N-7 MTase activities but completely abolished replication in an infectious clone [14].

### The interface modulates virus replication

Using biochemical and reverse genetic tools—enzymology of MTase and RdRp activities for DENV4 NS5, DENV4 replicon and DENV2 infectious virus, we examined the functional relevance of the interface interactions to viral RNA replication and infectivity by structure-guided mutagenesis. When either the MTase (Y119A and R263A (R262 in DENV3) in DENV4) or the polymerase activities (E270A in DENV4 (E269 in DENV3)) were severely inhibited (over 50% reduction compared to WT), neither viral RNA nor infectious virus could be observed (Fig. 5A; Table 2), Mutation of key polar contacts at the interface (K95A and R353A (R352 in DENV3)), did not impair the enzymatic activities with a slightly increased RdRp activities (Table 2). Indeed regulation of the RdRp *de novo* initiation activity by the MTase domain was elegantly demonstrated using an array of enzyme assays by Selisko and colleagues [33]. However, this enhancement in enzymatic activity was not beneficial for the virus: we observed a slightly delayed RNA replication as indicated by the reporter activity in the first 24 hrs in the replicon assay (Fig. 5A) and more severely impaired virus replication and infectivity in the infectious clone (Fig. 5B, C and D). For K95A, the intracellular and extracellular RNA levels was about 1 log lower with <50% virus recovery measured by plaque assay compared to WT. In the case of R353A, which had a two-fold higher elongation activity, there was no detectable virus replication. It is possible that the enhanced polymerase activities of mutants K95A or R353A may promote the production of incomplete genomes, truncated viral RNA fragments or catastrophic mutations that abolish reinfection. These mutations may also alter the NS5 conformational dynamics and affect its ability to form competent virus replication complex with other cofactors from the virus and the host. Taken together we conclude that the inter-domain interface of DENV NS5 is essential for successful virus replication and infectivity.

### An evolutionary view of the flavivirus NS5 MTase-RdRp structural heterogenity

Previous studies on WNV NS5 identified genetic interactions between residues K46A/R47A/E49A (MTase) and L512 (RdRp) from reverse genetic experiments, and a 3D model for WNV NS5 was built based on the hypothesis of direct molecular interactions [12]. However, mutagenesis studies targeting these residues do not affect the interaction between the two domains significantly using *in vivo* co-expression system [60]. The crystal structure of JEV NS5 revealed a hydrophobic inter-domain interface between the MTase and RdRp domains [31]. The follow-up mutagenesis study addressed the importance for JEV virus replication of residues P113 and L115 from the MTase domain and F351 (F348 in DENV3), F467 (F464 in DENV3) and P585 (P582 in DENV3) from the RdRp domain [64]. In the same study, corresponding mutations (P113D, P115D, F349D, F465D and to a lesser extent P583D) in DENV2 NS5 also yielded severely defective viruses [64]. The residues examined by mutagenesis involved drastic changes of the side-chain chemistry that may impact the folding and overall architecture of the mutants and limit the interpretations of these phenotypes. Subsequently in a follow-up work, the *in vitro* polymerase activity assays on JEV NS5 showed that proteins with mutations at the interface had only mildly impaired polymerase activities [65]. The authors thus suggested that the defect in virus replication was due to the amplifying effects of the defective enzymatic activity, the impact on the protein-protein interactions, or the combined effect of both [31,64,65].

Careful examination of the two available structures of NS5 revealed two non-overlapping buried surfaces on the MTase domain but one shared buried surface from the RdRp fingers subdomain (Figs. 1 and 3; S4 Fig in S1 Text) [31]. Indeed, F348 and P582 from DENV3 NS5 also form hydrophobic stacking interactions with the MTase domain (Fig. 1D and E). Mutations of these two residues, particularly F348, would disrupt the interface in both structures and the DENV3 NS5 structure explains very well the defective phenotypes of the mutant DENV2 viruses (F349D (F348 in DENV3) and P583D (P582 in DENV3)) (Figs. 1, 3 and 6) [64]. This further argues for the dynamic nature of NS5 and the differential impacts on virus replication within the replication complex machinery.

Based on the present report and comparable studies on NS5 from JEV and other flaviviruses, it is possible that the NS5 protein from different flaviviruses may have evolved to adopt different sets of conformations, stabilized by distinct interfaces and leading to different allosteric mechanisms to regulate their enzymatic activities (Figs. 3 and 6; S5 Fig in S1 Text). This hypothesis is detailed in Fig. 6. However, which molecular conformation(s) participates in the various stage(s) of the virus replication cycle remains elusive (Fig. 6). Thus, during virus replication, NS5 may adopt various conformations upon recruitment of and interaction with NS3, viral RNA and other viral and host cofactors, for instance in order to synthesize a fixed ratio of positive and negative RNA strands, for unwinding dsRNA intermediates and for the effective production of progeny viral genomic RNA. The short linker of DENV NS5 is likely to dictate the degree of freedom of the overall molecular conformation so that the necessary discrete functional conformations can be attained. Inspection of the sequence heterogeneity of the linker residues (Fig. 4E) suggests that the NS5 proteins from other flaviviruses may also be endowed with comparable (or even higher) degrees of freedom (Fig. 6), possibly reflecting the need to accommodate additional host factors that must be recruited to the RC for replication. This hypothesis is also indirectly supported by the disordered linker sequence of JEV NS5 (S3 Fig in S1 Text). Remarkably, the flaviviral NS3 protease-helicase also contains an inter-domain linker that is flexible and variable in sequence and which is able to adopt multiple conformations at different stages of the virus life cycle [66–70]. In this respect, the inter-domain linkers observed in the NS3 and NS5 proteins might have evolved to play a role for the assembly of the RC comparable to the extended and flexible arms that are needed for viral capsid proteins assembly. Further studies exploring possible connections between virus pathogenesis and the dynamics of viral replicative enzymes in various flaviviruses are now needed, which are likely to be of interest to both basic virology and immunology research and also to antiviral therapeutics development.

## Supporting Information

S1 Text S1 Fig. The final electron density maps of GTP-Mg^2+^ and SAH with Fourier coefficients 2mF_obs_-DF_calc_ are contoured at 1.0 σ (blue). S2 Fig. Deuterium uptake data for DENV3 NS5 and domains. Deuterium uptake data shown in the heat map format where peptides are represented using rectangular strips below the portion of the protein sequenc they map back to and colors are used to categorize average deuterium incorporation for each peptide. The key shows colors assigned to % deuterium ranges. (A) DENV3 NS5 (full length), (B) DENV3 NS5-MTase (1–296), (C) DENV3 NS5-RdRp (273–900). (D) Heat map data overlaid onto a crystal structure for the DENV3 NS5-MTase (1–296), and RdRp (273–900). The color key indicates the extent of the deuterium uptake (%), in which blue means the lowest deuterium uptake. Peptides that colored gray are regions that information of deuterium uptake is missing. F: motif F; G: motif G. (E) Comparison of HDX extent of the peptides at the inter-domain interface. Peptide F65–77, which positions as one interface region in MTase, exhibited similar deuterium uptake activities in both MTase protein (12%) and full-length NS5 protein (12%). Another interface region, residues K95–118, displayed 18% and 20% average deuterium uptakes for all HDX time points. (F) These two peptides displayed comparable HDX behavior in solutions as indicated by the same patterns of MS isotopic distributions. The C terminal region of MTase (peptide T243–273) also showed high hydrogen bonding activities in both MTase domain and NS5 samples. **S3 Fig**. Structure alignments of DENV3 NS5 with NS5-MTase and NS5-RdRp domains. (A) DENV3 NS5 is in purple, DENV3 NS5-MTase (PDB code: 3P97) is in yellow, DENV2 NS5-MTase (PDB code: 1L9K) in orange, DENV3 NS5-RdRp (PDB code: 4C11) in Cyan, DENV3 NS5-RdRp (2J7U) in gray. N’ and C’ stands for N-terminal and C-terminal of NS5 protein; G: motif G, residues 406–417; F: motif F, residues 455–468; Zn1 and Zn2 are two zinc cations in their binding sites. (B) Zoom-in view of the linker region. Key residues are displayed as sticks and the Cα atoms are shown as spheres. **S4 Fig**. Structure alignments of JEV full length NS5 with JEV RdRp domains. (A) JEV NS5 (PDB Code: 4K6M) is in red and JEV RdRp (PDB Code: 4HDG) is in blue. The structures are aligned and displayed as ribbon. Motifs F and G are shown as connected spheres and each sphere represents one residue. (B) Enlarged view at the domain linker region. **S5 Fig**. Homology modeling of flavivirus NS5 structures. (A) Homology modeling of DENV3, WNV, YFV, and TBEV NS5 to JEV NS5 was performed using HHpred server (http://toolkit.tuebingen.mpg.de/hhpred). The figure below is the structure alignment at the JEV interface. The hydrophobic residues at the interface are labeled based on DENV3 NS5. These residues are within the close proximity, suggesting a likely interface, possible for NS5 from all flaviviruses. (B) Structure alignment of the homology models of NS5 from JEV, WNV, YFV, and TBEV based on DENV3 NS5 crystal structure (this work), at the interface area. Similarly, the key polar residues that are labeled based on DENV3 NS5. These residues are also within the close proximity, suggesting a likely interface, possible for NS5 from all flaviviruses. **S1 Table**. List of DNA primers. (A) Primers used for site-directed mutagenesis (SDM) of DENV4-F plasmid. (B) Primers used for PCR of T7pro-LUC-NS3 cDNA cassette from pACYCI DENV4-WT replicon. (C) Primers used for PCR of NS5 full length mutant cDNA cassettes from pACYCI DENV4-F mutant Replicon plasmids (NheI,XhoI sites) into pET28a vector. (D) Primers used for site-directed mutagenesis (SDM) of DENV2 infectious clone. **S2 Table**. Summary of the MTase-RdRP interdomain interface analysis of NS5 from DENV3 and JEV. (A) Comparative analysis of MTase-RdRP in DENV3 NS5 and JEV NS5. The MTase domain and RdRP domain were defined as regions listed in the table. ^i^Nres: number of residues involved in MTase-RdRP interactions. Total buried area: calculated as the difference between total accessible surface areas of isolated and interfacing structures, divided by two, using PISA. Δ^i^G: Indicates the solvation free energy gain upon formation of the interface. The value is calculated as difference in total solvation energies of isolated and interfacing structures, using PISA. *N*HB/*N*SB/*N*vdW: number of putative hydrogen bonds, salt bridges and additional van der Waals interactions that contribute to the interface. (B) Pairs of residues from MTase and RdRp that are involved in inter-domain interactions.
(DOCX)

S1 Movie Modelling the possible conformational transitions of MTase-RdRp. Initial state was based on JEV NS5 and the end state was based on DENV3 NS5 structure.
(MPG)

S2 Movie Consurf analysis of DENV3 NS5. MTase domain is displayed as cartoon and the RdRp domain is in surface view. Same color theme as in Fig. 2A.
(MPG)

S3 Movie Consurf analysis of JEV NS5. MTase domain is displayed as cartoon and the RdRp domain is in surface view. Same color theme as in Fig. 2A.
(MPG)
