# Supplementary material for: A Crystal Structure of the Dengue Virus NS5 Protein Reveals a Novel Inter-domain Interface Essential for Protein Flexibility and Virus Replication
Source: PLoS Pathog. 2015 Mar 16;11(3):e1004682. doi: 10.1371/journal.ppat.1004682 (PMC4361662; doi:10.1371/journal.ppat.1004682)
Supplement: S1 Text — Deuterium uptake data shown in the heat map format where peptides are represented using rectangular strips below the portion of the protein sequenc they map back to and colors are used to categorize average deuterium incorporation for each peptide. The key shows colors assigned to % deuterium ranges. (A) DENV3 NS5 (full length), (B) DENV3 NS5-MTase (1–296), (C) DENV3 NS5-RdRp (273–900). (D) Heat map data overlaid onto a crystal structure for the DENV3 NS5-MTase (1–296), and RdRp (273–900). The color key indicates the extent of the deuterium uptake (%), in which blue means the lowest deuterium uptake. Peptides that colored gray are regions that information of deuterium uptake is missing. F: motif F; G: motif G. (E) Comparison of HDX extent of the peptides at the inter-domain interface. Peptide F65–77, which positions as one interface region in MTase, exhibited similar deuterium uptake activities in both MTase protein (12%) and full-length NS5 protein (12%). Another interface region, residues K95–118, displayed 18% and 20% average deuterium uptakes for all HDX time points. (F) These two peptides displayed comparable HDX behavior in solutions as indicated by the same patterns of MS isotopic distributions. The C terminal region of MTase (peptide T243–273) also showed high hydrogen bonding activities in both MTase domain and NS5 samples. S3 Fig. Structure alignments of DENV3 NS5 with NS5-MTase and NS5-RdRp domains. (A) DENV3 NS5 is in purple, DENV3 NS5-MTase (PDB code: 3P97) is in yellow, DENV2 NS5-MTase (PDB code: 1L9K) in orange, DENV3 NS5-RdRp (PDB code: 4C11) in Cyan, DENV3 NS5-RdRp (2J7U) in gray. N’ and C’ stands for N-terminal and C-terminal of NS5 protein; G: motif G, residues 406–417; F: motif F, residues 455–468; Zn1 and Zn2 are two zinc cations in their binding sites. (B) Zoom-in view of the linker region. Key residues are displayed as sticks and the Cα atoms are shown as spheres. S4 Fig. Structure alignments of JEV full length NS5 with JEV RdRp domains. (A) J [file ppat.1004682.s001.docx]

**Supplementary Information**

**A crystal structure of the dengue virus NS5 protein reveals a novel inter-domain interface essential for protein flexibility and virus replication**

Yongqian Zhao ^1,2^, Tingjin Sherryl Soh ^3,6^, Jie Zheng ^3^, Kitti Wing Ki Chan ^1^, Wint Wint Phoo ^4^, Chin Chin Lee ^1^, Moon YF Tay ^1^, Kunchithapadam Swaminathan ^2,5^, Tobias C. Cornvik ^3^, Siew Pheng Lim ^6^, Pei-Yong Shi ^6^, Julien Lescar ^3,7*^, Subhash G. Vasudevan ^1,2*^, Dahai Luo ^4*^

^1^Program in Emerging Infectious Diseases, DUKE-NUS Graduate Medical School, 8 College Road, Singapore 169857

^2^NUS Graduate School for Integrative Sciences and Engineering, National University of Singapore, 28 Medical Drive, Singapore 117456

^3^School of Biological Sciences, Nanyang Technological University. 60 Nanyang Drive, Singapore 637551

^4^Lee Kong Chian School of Medicine, Nanyang Technological University, 61 Biopolis Drive, Proteos Building, #07-03, Singapore 138673

^5^Department of Biological Sciences, National University of Singapore, Singapore 117543

^6^Novartis Institute for Tropical Diseases, 10 Biopolis Road, Singapore 138670

^7^UPMC UMRS CR7 - CNRS ERL 8255-INSERM U1135 Centre d’Immunologie et des Maladies Infectieuses. Centre Hospitalier Universitaire Pitié-Salpêtrière, Faculté de Médecine Pierre et Marie Curie, Paris, France

**Supplementary Figures**

**Figure S1.** The final electron density maps of GTP-Mg^2+^ and SAH with Fourier coefficients 2mF_obs_-DF_calc_ are contoured at 1.0 σ in blue mesh.


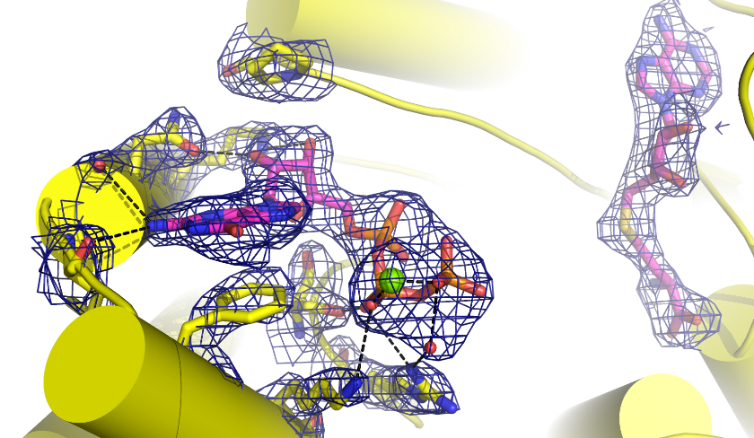


**Figure S2. Deuterium uptake data for DENV3 NS5 and domains.** Deuterium uptake data shown in heat map format where peptides are represented using rectangular strips below the portion of the protein sequence they map back to and colors are used to categorize average deuterium incorporation for each peptide. The Key shows colors assigned to the deuterium ranges (%). (A) DENV3 NS5, (B) DENV3 MTase (1-296), (C) DENV3 RdRP (273-900). (D) Heat map data overlaid onto a crystal structure for the NS5-MTase and NS5-RdRp domains. The color key indicates the extent of the deuterium uptake, in which blue means the lowest deuterium uptake. Peptides in gray are regions that information of deuterium uptake is missing. (E) Comparison of HDX extent of the peptides at the inter-domain interface. Peptide F65-77, which positions as one interface region in MTase, exhibited similar deuterium uptake activities in both MTase protein (12%) and full-length NS5 protein (12%). Another interface region, residues K95-118, displayed 18% and 20% average deuterium uptakes for all HDX time points. (F) These two peptides displayed comparable HDX behavior in solutions as indicated by the same patterns of MS isotopic distributions. The C terminal region of MTase (peptide T243-273) also showed high hydrogen bonding activities in both MTase domain and NS5 samples.

**(A)**

**
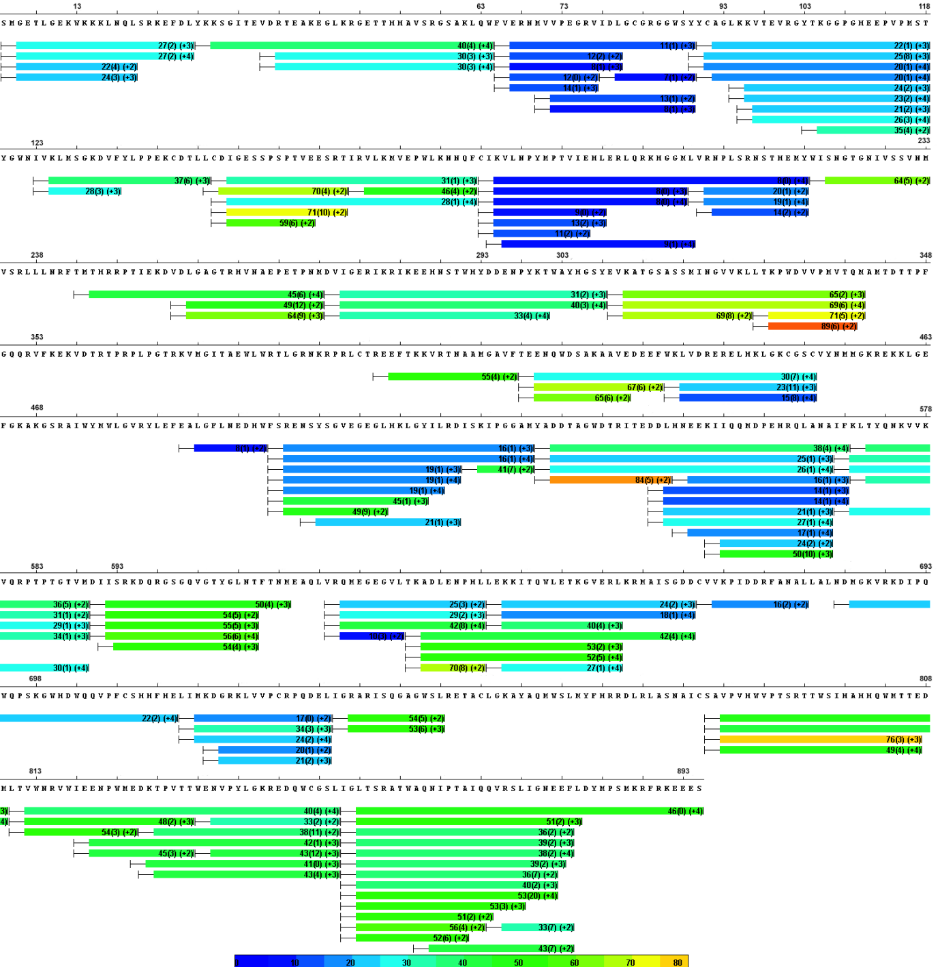
**

**(B)**


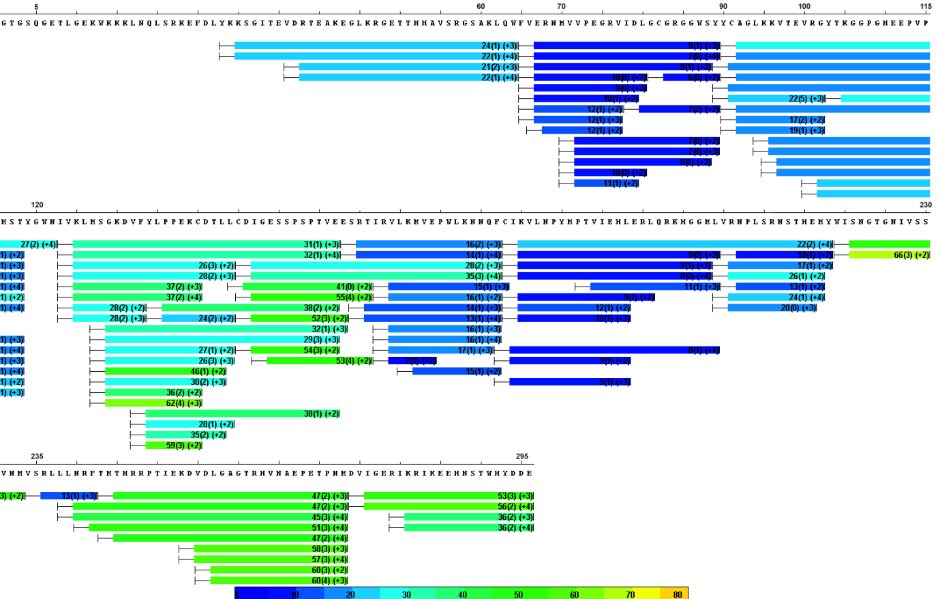


**(C)**


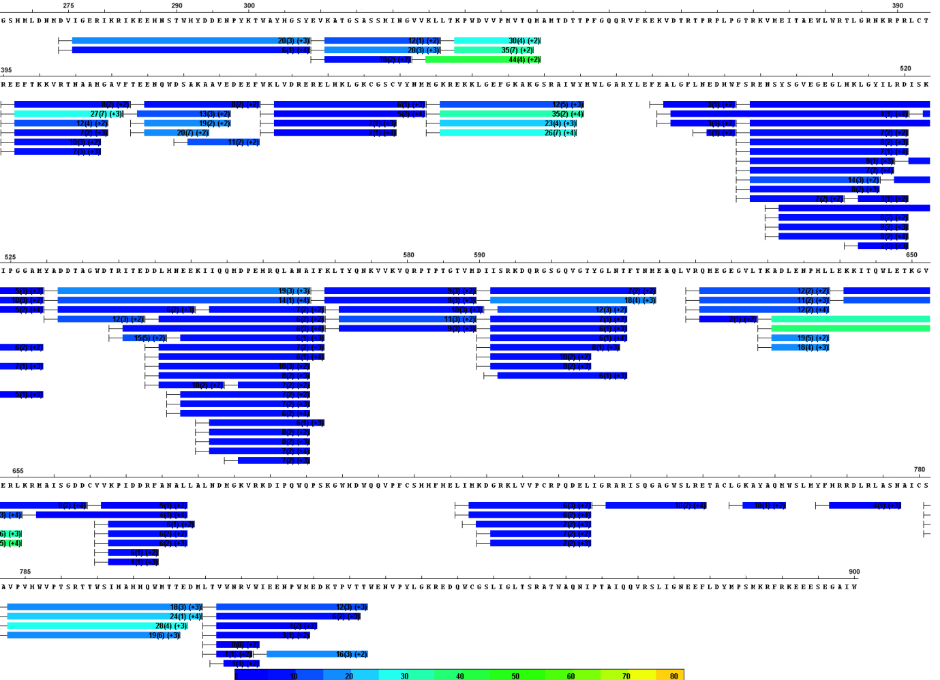


**(D)**

**
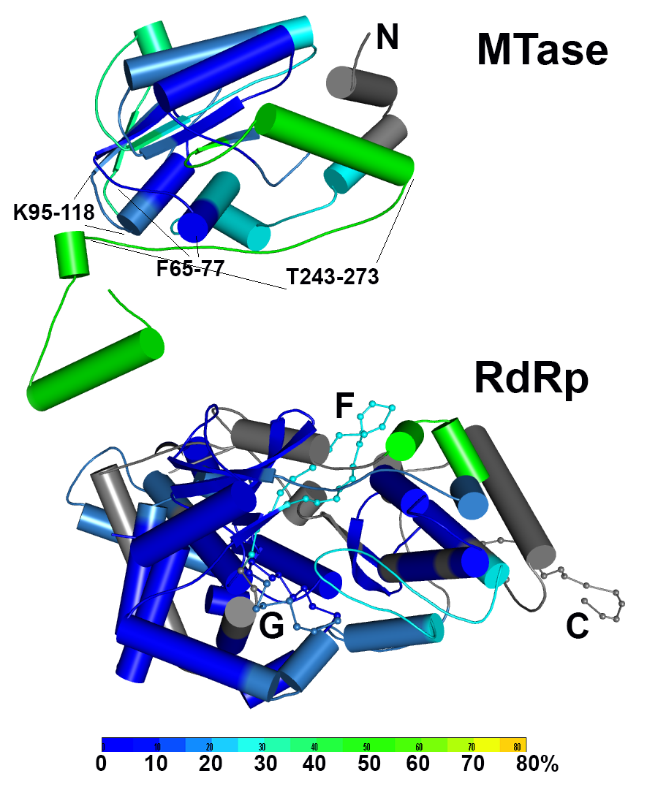
**


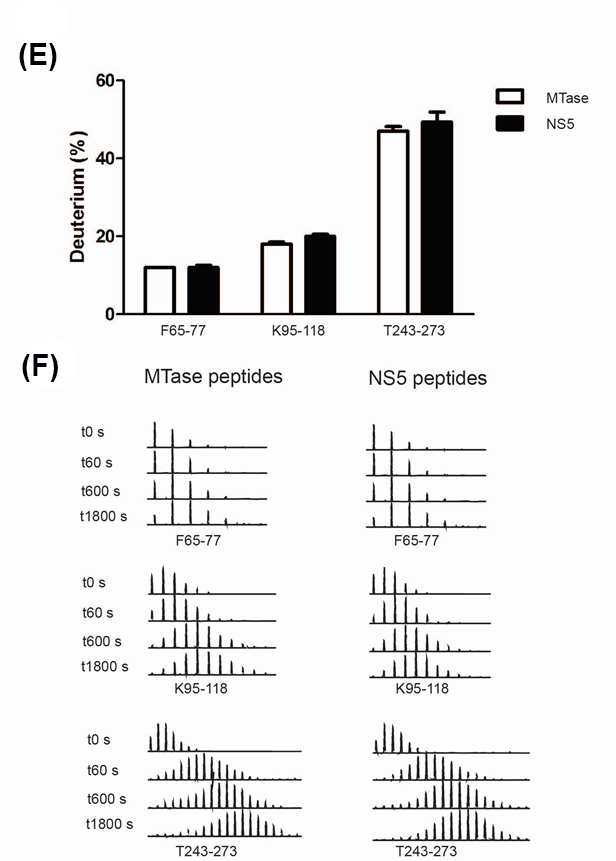


**Figure S3. Structure alignments of DENV3 NS5FL with NS5-MTase and NS5-RdRp domains. (A)** DENV3 NS5FL is in purple, DENV3 NS5-MTase (PDB code: 3P97) is in yellow, DENV2 NS5-MTase (PDB code: 1L9K) in orange, DENV3 NS5-RdRp (PDB code: 4C11) in Cyan, DENV3 NS5-RdRp (2J7U) in gray. N’ and C’ stands for N-terminal and C-terminal of NS5 protein; G: motif G, residues 406-417; F: motif F, residues 455-468; Zn1 and Zn2 are two zinc cations in their binding sites. (B) Zoom-in view of the linker region. Key residues are displayed as sticks and the Cα atoms are shown as spheres**.**

**
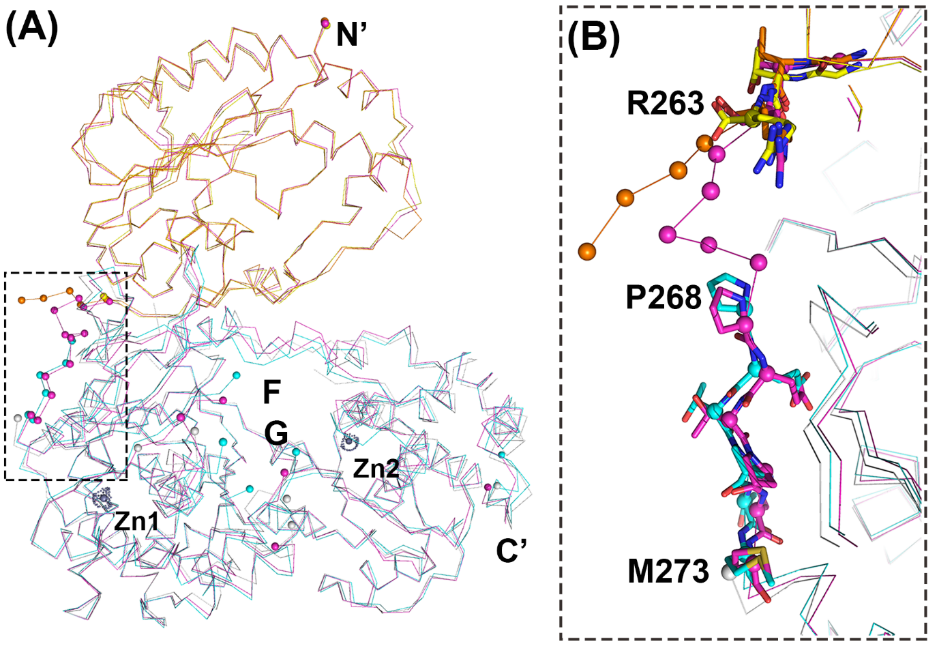
**

**Figure S4. Structure alignments of JEV full length NS5 with JEV RdRp domains. (A)** JEV NS5FL (PDB Code: 4K6M) is in red and JEV RdRp (PDB Code: 4HDG) is in blue. The structures are aligned and displayed as ribbon. Motifs F and G are shown as connected spheres and each sphere represents one residue. (B) Enlarged view at the domain linker region.

**
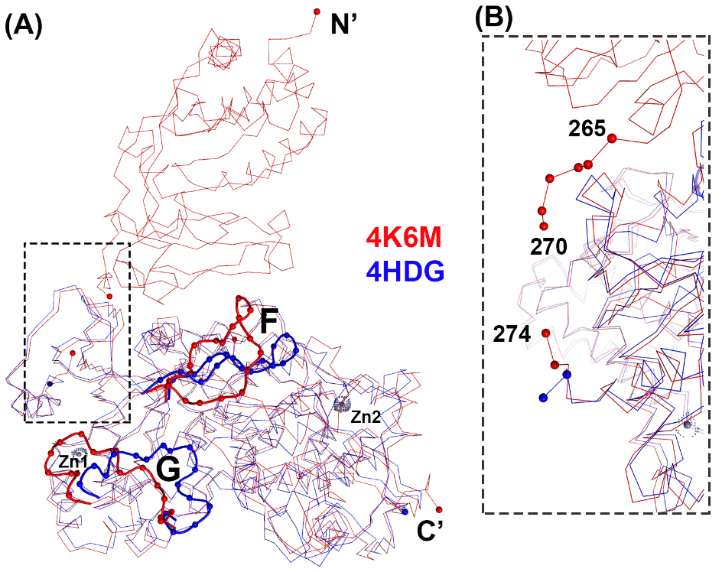
**

**Figure S5 Homology modeling of flavivirus NS5 structures.** **(A)** Homology modeling of DENV3, WNV, YFV, and TBEV NS5 to JEV NS5 has been performed using HHpred server (<http://toolkit.tuebingen.mpg.de/hhpred>). The figure below is the structure alignment at the JEV interface. The hydrophobic residues at the interface are labeled based on DENV3 NS5. These residues are within the close proximity, suggesting a likely interface, possible for NS5 from all flaviviruses. **(B)** Structure alignment of the homology models of NS5 from JEV, WNV, YFV, and TBEV based on DENV3 NS5 crystal structure, at the interface area. Similarly, the key polar residues that are labeled based on DENV3 NS5. These residues are also within the close proximity, suggesting a likely interface, possible for NS5 from all flaviviruses.

**(A)**

**
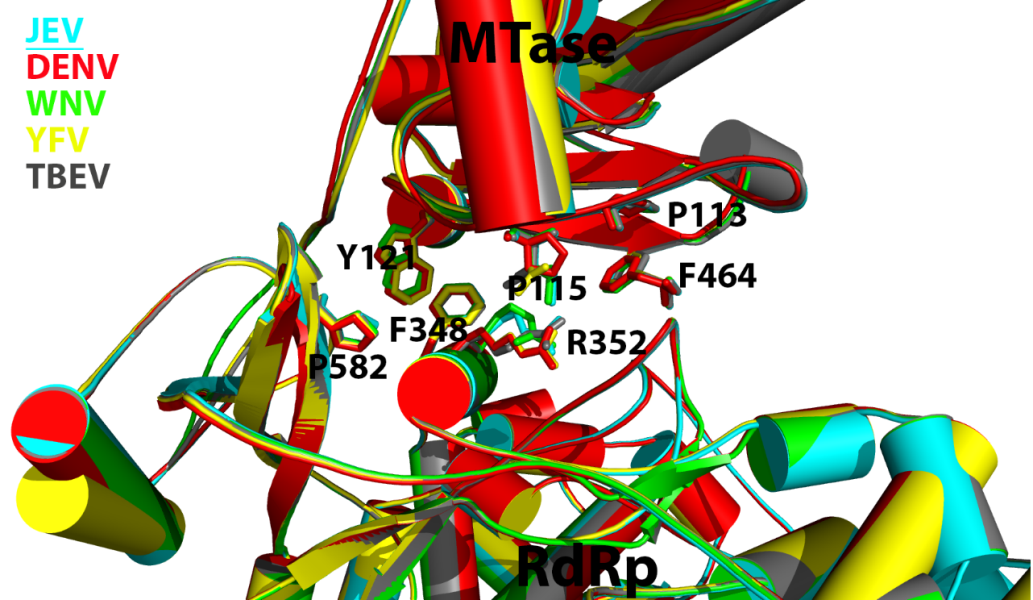
**

**(B)**


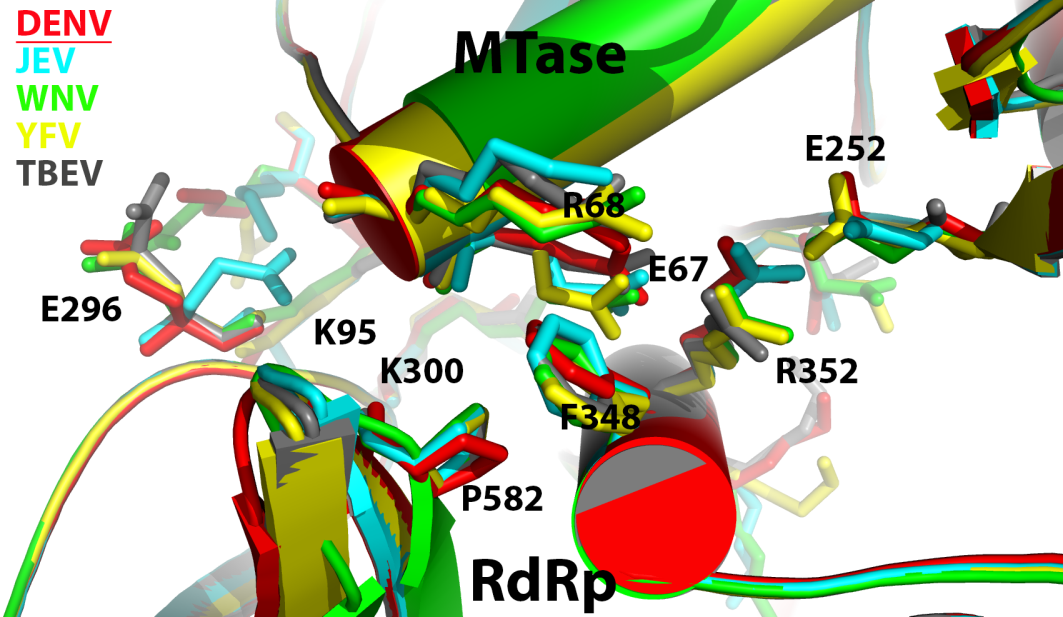


**Supplementary Tables**

**Table S1. List of DNA primers.**

1. Primers used for site-directed mutagenesis (SDM) of DV4-F plasmid

| Mutation(s)*^a^* | Primer | | |
| --- | --- | --- | --- |
|  | **Orientation*^c^*** | | **Sequence** |
| K95A | For | GTCCTATTACATGGCGACACTCGCAAACGTGACTGAAGTGAAAGGAT | |
|  | Rev | ATCCTTTCACTTCAGTCACGTTTGCGAGTGTCGCCATGTAATAGGAC | |
|  |  |  | |
| Y119A | For | ATGAAGAACCAATCCCCATGGCTACTGCAGGCTGGAATTTGGTCA | |
|  | Rev | TGACCAAATTCCAGCCTGCAGTAGCCATGGGGATTGGTTCTTCAT | |
|  |  |  | |
| R263A | For | GATCTTGGGGCAGGAACGGCAAGTGTCTCCACTGAAAC | |
|  | Rev | GTTTCAGTGGAGACACTTGCCGTTCCTGCCCCAAGATC | |
|  |  |  | |
| E268A | For | CGAGAAGTGTCTCCACTGCAACAGAAAAACCAGACAT | |
|  | Rev | ATGTCTGGTTTTTCTGTTGCAGTGGAGACACTTCTCG | |
|  |  |  | |
| E270A | For | GAAGTGTCTCCACTGAAACAGCAAAACCAGACATGACAATTAT | |
|  | Rev | ATAATTGTCATGTCTGGTTTTGCTGTTTCAGTGGAGACACTTC | |
|  |  |  | |
|  |  |  | |
| R353A | For | CAACCCCTTTTGGGCAACAAGCAGTGTTCAAGGAGAAGGT | |
|  | Rev | ACCTTCTCCTTGAACACTGCTTGTTGCCCAAAAGGGGTTG | |

*^a^* Mutations generated using QuikChange II XL SDM kit.

*^b^* Double mutation generated using QuikChange multi SDM kit.

*^c^* For, forward; Rev, reverse.

1. Primers used for PCR of T7pro-LUC-NS3 cDNA cassette from pACYCI DV4-WT replicon

| Primer | Orientation*^a^* | Sequence |
| --- | --- | --- |
| DV4-Rep-NotI-FOR | For | GTATCACATATTCTGCGGC |
| DV4-Rep-KpnI-REV | Rev | ATCCATCACTATGAGGTTAT |

*^a^* For, forward; Rev, reverse.

1. Primers used for PCR of NS5 FL mutant cDNA cassettes from pACYCI DV4-F plasmids

| Primer | Orientation*^a^* | Sequence*^b^* |
| --- | --- | --- |
| D4 FL-NheI-FOR | For | GCTAGCTAGCGGAACTGGGACCACAGGAG |
| D4 FL-XhoI-REV | Rev | CTCACTCGAGTTACAGAACTCCTTCACTCT |

*^a^* For, forward; Rev, reverse.

*^b^* Restriction enzyme sequences are underlined.

1. Primers used for site-directed mutagenesis (SDM) to construct mutant DV2 infectious clones

| Mutation(s)*^a^* | Primer | | |
| --- | --- | --- | --- |
|  | **Orientation*^b^*** | | **Sequence** |
| K95A | For | GGTCATACTATTGTGGGGGACTAGCGAATGTAAAAGAAGTCAAAGGCC | |
|  | Rev | GGCCTTTGACTTCTTTTACATTCGCTAGTCCCCCACAATAGTATGACC | |
|  |  |  | |
| Y119A | For | CCTATTCCCATGTCAACAGCCGGTTGGAATCTGGTGCGTCTTCAAAGTGG | |
|  | Rev | CCACTTTGAAGACGCACCAGATTCCAACCGGCTGTTGACATGGGAATAGG | |
|  |  |  | |
| E268A | For | GGAACTCGCAATATCGGAATTGCAAGTGAGACACCAAATTTAGAC | |
|  | Rev | GTCTAAATTTGGTGTCTCACTTGCAATTCCGATATTGCGAGTTCC | |
|  |  |  | |
| R353A | For | CGACTCCATTTGGACAACAGGCCGTTTTCAAAGAGAAGGTGGACACG | |
|  | Rev | CGTGTCCACCTTCTCTTTGAAAACGGCCTGTTGTCCAAATGGAGTCG | |

*^a^* Mutations generated using QuikChange II XL SDM kit.

*^b^* For, forward; Rev, reverse.

**Table S2. Summary of the MTase-RdRP interdomain interface analysis of NS5 from DENV3 and JEV. (A)** Comparative analysis of MTase-RdRP in DENV3 NS5 and JEV NS5. The MTase domain and RdRP domain were defined as regions listed in the table. ^i^Nres: number of residues involved in MTase-RdRP interactions. Total buried area: calculated as the difference between total accessible surface areas of isolated and interfacing structures, divided by two, using PISA. Δ^i^G: Indicates the solvation free energy gain upon formation of the interface. The value is calculated as difference in total solvation energies of isolated and interfacing structures, using PISA. *N*HB/*N*SB/*N*vdW: number of putative hydrogen bonds, salt bridges and additional van der Waals interactions that contribute to the interface. **(B)** Pairs of residues from MTase and RdRp that are involved in inter-domain interactions.

**(A)**

|  | DENV3 NS5 | | JEV NS5 | |
| --- | --- | --- | --- | --- |
|  | MTase | RdRp | MTase | RdRp |
| Residues | 6-262 | 273-883 | 5-265 | 276-895 |
| ^i^Nres | 26 | 23 | 21 | 19 |
| Interface area, Å² | 726. 3 | 775. 9 | 713. 0 | 751. 0 |
| Total Buried Area, Å² | **1502.2** | | **1464** | |
| gain on complex formation, Δ^i^G, kcal/mol | 2.4 | | -6.9 | |
| average gain, kcal/mol | -3.1 | | -1.4 | |
| P-value | 0. 845 | 0. 780 | 0. 151 | 0. 162 |
| NHB / NSB | 9 / 8 | | 10 / 3 | |
| NvdW | 5 | 4 | 7 | 6 |

**(B)**

| **MTase/ RdRP residue in DENV3 NS5** | **Interaction type*** | **MTase/ RdRP residue in JEV NS5** | **Interaction type*** |
| --- | --- | --- | --- |
| E67/R352 | 2HB/4SB | R101/E359 | 2HB/3SB |
| K95/N297 | HB | Y119/E299 | HB |
| K95/Y299 | HB | W121/K358 | HB |
| K96/E296 | HB/SB | N122/K358 | HB |
| E252/R352 | 2HB/2SB | V124/K358 | HB |
| D256/E356 | HB | S125/E359 | HB |
| D256/K357 | HB/SB | S262/E588 | HB |
| W64/R68/F348/P582 | Hydrophobic interactions | T264/E299 | HB |
|  |  | T264/R584 | HB |
|  |  | P113/L115/W121/P351/F467/P585 | Hydrophobic interactions |

**HB: Hydrogen bond; SB: Salt bridge*

**Supplementary Moive 1.** Modelling the possible conformational transitions of MTase-RdRp. Initial state was based on JEV NS5 and the end state was based on DENV3 NS5 structure.

**SUPPLEMENTAEY MOVIE 2.** **Consurf analysis of DENV3 NS5.** MTase domain is displayed as cartoon and the RdRp domain is in surface view. Same color theme as in Figure 2A.

**SUPPLEMENTAEY MOVIE 3.** **Consurf analysis of JEV NS5.** MTase domain is displayed as cartoon and the RdRp domain is in surface view. Same color theme as in Figure 2A.
